# The genome of *Magnolia hypoleuca* provides a new insight into cold tolerance and the evolutionary position of magnoliids

**DOI:** 10.3389/fpls.2023.1108701

**Published:** 2023-02-10

**Authors:** Luojing Zhou, Feixia Hou, Li Wang, Lingyu Zhang, Yalan Wang, Yanpeng Yin, Jin Pei, Cheng Peng, Xiaobo Qin, Jihai Gao

**Affiliations:** ^1^ State Key Laboratory of Southwestern Chinese Medicine Resources, Chengdu University of Traditional Chinese Medicine, Chengdu, China; ^2^ Sichuan Academy of Forestry Sciences, Chengdu, China; ^3^ Sichuan Provincial Academy of Natural Resource Sciences, Chengdu, China; ^4^ School of Preclinical Medicine, Chengdu University, Chengdu, China

**Keywords:** Magnoliaceae, *Magnolia hypoleuca*, cold tolerance, floral scent, evolutionary, comparative genomics

## Abstract

*Magnolia hypoleuca Sieb. & Zucc*, a member of the Magnoliaceae of magnoliids, is one of the most economically valuable, phylogenetic and ornamental tree species in Eastern China. Here, the 1.64 Gb chromosome-level assembly covers 96.64% of the genome which is anchored to 19 chromosomes, with a contig N50 value of 1.71 Mb and 33,873 protein-coding genes was predicted. Phylogenetic analyses between *M. hypoleuca* and other 10 representative angiosperms suggested that magnoliids were placed as a sister group to the eudicots, rather than sister to monocots or both monocots and eudicots. In addition, the relative timing of the whole-genome duplication (WGD) events about 115.32 Mya for magnoliid plants. *M. hypoleuca* was found to have a common ancestor with *M. officinalis* approximately 23.4 MYA, and the climate change of OMT (Oligocene-Miocene transition) is the main reason for the divergence of *M. hypoleuca* and *M. officinalis*, which was along with the division of Japanese islands. Moreover, the *TPS* gene expansion observed in *M. hypoleuca* might contribute to the enhancement of flower fragrance. Tandem and proximal duplicates of younger age that have been preserved have experienced more rapid sequence divergence and a more clustered distribution on chromosomes contributing to fragrance accumulation, especially phenylpropanoid, monoterpenes and sesquiterpenes and cold tolerance. The stronger selective pressure drived the evolution of tandem and proximal duplicates toward plant self-defense and adaptation. The reference *M. hypoleuca* genome will provide insights into the evolutionary process of *M. hypoleuca* and the relationships between the magnoliids with monocots and eudicots, and enable us to delve into the fragrance and cold tolerance produced by *M. hypoleuca* and provide more robust and deep insight of how the Magnoliales evolved and diversified.

## 1 Introduction

The Magnoliaceae, comprises two genera, *Liriodendron* L and *Magnolia* L, is an important plant resource for landscaping, medicine and timber, and has high economic value and ecological benefits (Endress, 2010; Romanov and Dilcher, 2013; Li et al., 2020). Magnoliaceae have large and gorgeous flowers, fragrant flowers with different life forms, trees and shrubs, evergreen and deciduous. *Magnolia officinalis (M. officinalis)* and *Magnolia biondii* (*M. biondii)* have been extensive research in phytochemical and pharmacological and have wide range of lignan bioactive compounds, with at least 255 different reported components, such as lignans, neolignans, phenylpropanoids, and terpenes (Lee et al., 2011; Morshedloo et al., 2017; Sarrica et al., 2018; Zhang et al., 2021). The Magnoliaceae plant has been used as a traditional herb for thousands of years in Asia, and its bark and flowers are used in traditional Chinese and Japanese medicine to treat gastrointestinal disorders, anxiety and allergic diseases (Lee et al., 2011; Ranaware et al., 2018). The origin of Magnoliaceae may be in Southwest China, which may also be a differentiation and diversification center, and it radiates outward and enters North America through Japan, Russia and the Far East (Liu et al., 1995). The western limit of the distribution of this family is southeast Himalayas (including northeast India), extending northeast to Japan, which is also the northernmost limit of the distribution of this family, at about 45° N latitude, that is, the Chiba Islands of Japan, south to New Guinea and Brazil in the southern hemisphere (Azuma et al., 2001; Zhang, 2001). Magnoliaceae plants are considered to be the primitive class group in angiosperms due to the original characteristics, and there are great disputes on the determination of genus boundaries and species (Teng, 2010; Zeng et al., 2014; Liu et al., 2015). The phylogenetic position of magnoliids relative to monocotyledons and eudicots remains to be discussed. There are three general theories for phylogenetic position that magnoliids are resolved as sister to eudicots, magnoliids are resolved as sister to monocots, and magnoliids are resolved as sister to the clade of eudicots and monocots. For example, mang magnoliales genomes [*M. biondii* (Dong et al., 2021), and *M. officinalis* (Yin et al., 2021)*, Persea americana* (Mán et al., 2019)*, Annona muricata* (Strijk et al., 2021)*, Liriodendron chinense* (Chen et al., 2019)*, Chimonanthus salicifolius* (Lv et al., 2020)*, Chimonanthus praecox* (Shang et al., 2020)] have been subsequently published to reveale the phylogenetic positions were different. These studies have important phylogenetic implications for a deeper insight of the evolution of existing flowering plants.

**Figure 1:**
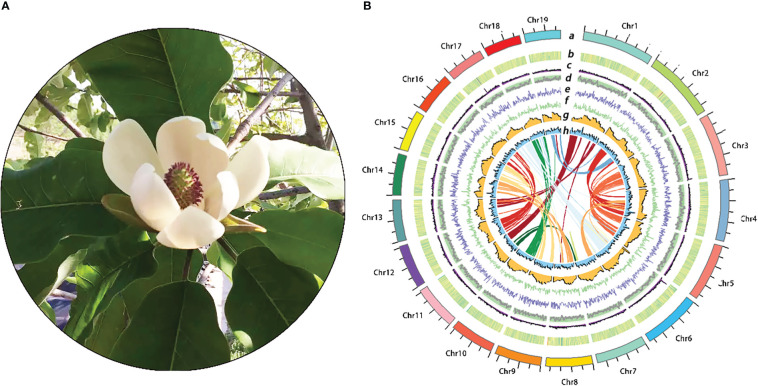
Characterization and features of the turmeric genome. **(A)**: Images of *M. hypoleuca*. **(B)**: The Distribution of *M. hypoleuca* genome features. **(a)** The 19 chromosomes, **(b)** guanine- cytosine (GC) density, **(c)** gene density, **(d)** LTR Assembly Index (LAI) density, **(e)** Copia density, **(f)** Gypsy density, **(g)** LTR density, **(h)** Full-LTR density, and Intragenomic synteny regions.

*Magnolia hypoleuca* Sieb. & Zucc (modern name: *Magnolia obovate* Thunb) is a member of the Magnoliaceae family and native to Hokkaido, Japan with name of Hoogashiwa and has been introduced into China in the thirties and forties in 20th century (**Figure 1A**) (Kinoshita, 2018). The bark extracts of *M. hypoleuca* and *M. officinalis* have been known for thousands of years in traditional Chinese and Japanese medicine and are still used extensively in herbal preparations with sedative, antioxidant, anti-inflammatory, antibiotic and anti-spasmodic functions (Lee et al., 2011). Previous studies have shown that the flower extract of *M. hypoleuca* and *M. officinalis* in flower extract were the same function with bark samples (Lee et al., 2018; Ham et al., 2020; Lovecká et al., 2020; Jhun et al., 2020). *M. hypoleuca* trees are valued by landscape gardeners and wood is used for furniture and various industrial arts. At present, *M. hypoleuca* is only native of Japan, other places such as China, Korea are introduced because of the broad development prospects with landscaping ornamental and economic value (Hori et al., 2019). One of the most beautiful of medicinal magnoliaceae plants, *M. hypoleuca* is also quite resistance to cold. *M. hypoleuca* grows in low temperature environment and has many biological traits superior to other magnoliaceae, such as its cold tolerance, its growth rate and maturity rate are faster than similar plants, especially in the young growth period, at a rate of 60~90 cm per year growth, is an ideal material for analyzing and breeding magnolia species (Kwon and Oh, 2015; Oguchi et al., 2017). Therefore, the intense fragrance and cold tolerance characters are the attractive features of *M. hypoleuca* which is also different from *M. officinalis*. First of all, floral fragrances are composed of specific plant metabolites that facilitate plant-environment interactions, attract pollinators and protect themselves from pathogens, herbivores etc (Matsuki et al., 2008). Schiestl found that terpenes and phenylpropanoid compounds are the main substances that emit signals to attract pollinators, while aliphatic compounds mainly play a defensive role between plants and herbivores (Schiestl, 2010). The main components of floral fragrances are a mixture of volatile organic compounds (VOCs), including terpenes, phenylpropanoids and fatty acid derivatives (Dudareva and Pichersky, 2000), which are also rich in Magnolia species flowers (Zhao, 2005). Monoterpenes and sesquiterpene which are usually generated *via* the 2-C-methyl-D-erythritol 4-phosphate (MEP) pathway and the mevalonate (MVA) pathway, respectively, are the major components of floral volatile organic compounds in Magnolia. The *TPS*s are key enzymes responsible for the last catalytic reaction in the MVA and MEP pathway and generate the final terpenoid compounds from different precursors. Currently, the molecular mechanisms and regulation of stronger floral fragrances compared with *M. hypoleuca* are not clear. On the other hand, the plant growth, development and geographical distribution are also influenced by low temperatures which are the main limiting factors for magnoliaceae to be introduced to northern high latitudes (Shi et al., 2015; Shi et al., 2018; Ding et al., 2019; Ding et al., 2020). Studies have shown that after receiving the low temperature signal, plants start the defense mechanism, increase the permeability of cell membrane, enhance the activity of protective enzymes, produce reactive oxygen species and accumulate the contents of osmoregulatory factors, while the biochemical pathways related to metabolism will be inhibited (Eric et al., 2010; Rudi et al., 2011; Xiao et al., 2018). In addition, low temperature freezing injury is a frequent natural disaster in agricultural production and landscaping, which not only limits the geographical distribution of crops and garden tree species, but also seriously affects the quality and yield of crops and the application of garden tree species (Strimbeck et al., 2015). Therefore, the study on the cold resistance physiology and molecular mechanism of *M. hypoleuca* under low temperature will not only provide excellent reserve genes and theoretical basis for more systematic and in-depth study of cold resistance related regulatory genes of Magnoliaceae plants and molecular breeding of cold resistant varieties in the future, but also have important practical significance for further introduction and cultivation of this species to high latitude areas.

*M. hypoleuca* has been shown to have a close genetic relationship with *M. officinalis* and *M. officinalis* Rehd. & Wils. ssp. biloba (Rehd. & Wils) Law in our previous study (Zhang et al., 2022), and it has different biological features compared to the latter two such as strong cold resistance. In recent years, the research on the biosynthetic pathway of flower fragrance and its material components, the mutual regulation of multiple genes under low temperature environment, and the physiological and biochemical functions of plants has been gradually deepened. At present, hundreds of genes have been found to be associated with the biosynthetic pathway of flower fragrance and cold resistance genetic engineering that has become a new hotspot in plant research (Spitzer-Rimon et al., 2010; Chan et al., 2016; Zhang et al., 2020). As an important means to study plant gene function, high throughput sequencing technology widely used in the study of low temperature response mechanism, molecular mechanism of volatile organic compound biosynthesis and regulation of *Arabidopsis thaliana* (Jiang et al., 2021), *Olea europaea* (Rao et al., 2021) and *C. praecox* (Shang et al., 2020; Shen et al., 2021). In addition, the limited genetic resources of *M. hypoleuca* have hindered the progress of research on the aromatic biosynthetic metabolism and cold resistance mechanisms of Magnoliaceae. The genome sequence of *M. hypoleuca* is important for molecular breeding, germplasm conservation and scientific research.

## 2 Results

### 2.1 Genome sequencing and assembly summary

The genomic DNA of *M. hypoleuca* was extracted from fresh leaves of a mature tree collected in northeast China and 57-fold high-quality sequence was generated form Illumina sequencing (97G), 170-fold Oxford Nanopore sequencing (~288G), and 50-fold Hi-C data (~85G) (**Supplementary Table S1**). The assembled genome of ONT sequence was 1.64G including 769 contigs with N50 read length of 8.65 Mb (**Supplementary Table S2**). The Hi-C library was constructed and sequenced to further employ assemble the contigs into 19 pseudochromosomes, with a total size of 1.64Gb genome sequences by using ALLHIC (Zhang et al., 2019) v0.9.8. The lengths of the 19 *M. hypoleuca* pseudochromosomes ranged from 69.1 Mb to 135.1 Mb (**Supplementary Table S3**, **Supplementary Figure S1**), and the N50 of chromosome were 88.9 Mb (**Supplementary Table S4**).

For genome quality assessment, The benchmarking universal single-copy orthologs (BUSCO) analysis identified that 98.6% of the completeness of the BUSCO gene set were ‘Complete Single-Copy BUSCOs’ in the *M. hypoleuca* genome (including 95.7% complete single-copy genes and 2.9% complete duplicated genes), while 0.7% were ‘Missing BUSCOs’. These results allow to obtain an improvement in quality compared to the unassembled chromosome level (**Supplementary Table S5**). Besides, we compared the Illumina short read sequences with the assembled genome and showed a mapping rate of 99.3%. Taken together, the above results indicated that there was high level of the contiguity, completeness and accuracy of the *M. hypoleuca* genome.

### 2.2 Genome annotations

Based combinatorial gene prediction strategy, considering evidence from *de novo* prediction, homology-based prediction, and transcriptome-based prediction, A total of 1,142,897,716 bp (64.54% of the whole *M. hypoleuca* genome) bases of repetitive sequences were identified in *M. hypoleuca*. LTR elements were the major repeat type, accounting for 57.63% of the *M. hypoleuca* genome length (**Supplementary Table S6**). Copia and Gypsy elements which is the two LTR superfamily elements were 307,794,727 and 433,746,899 bp, respectively, and accounting for 17.38% and 24.49% of the LTR repeat length, respectively. The results that LTR/Copia has a lower rate than LTR/Gypsy were similar to other magnoliids. The density of Gypsy elements shows a negative correlation with gene density, that is it decreases as gene density increases, while Copia elements are scattered in the genome, so there is no obvious pattern with the gene density distribution. The gene density of chromosomes ranges from 0-120 genes per megase, with uneven spatial distribution along the chromosome and high density in the middle of the chromosome arms (**Figure 1B**). Although the density of protein-coding genes appears to be generally complementary to repetitive elements across the genome, we found that long genes larger than 20 kb are more inclined to be distributed in duplicated regions (**Figure 1B**). DNA transposons and simple repeats constituted 25,962,439 and 21,669,688 bp accounting for 1.47% and 1.22%, respectively, of the genome length.

For gene prediction, the populations of 33,873 protein-coding gene models with an average length of 340 aa and coding DNA sequences (CDSs) with an average length of 1021 bp were predicted, comparable to that in *M. biondii* and *Oryza sativa* (**Supplementary Table S7**). Of the 33,873 genes, 30,858 (91.1%) were indicated by other species homology identification and RNA-seq data. A total of 2749 non-coding RNAs (ncRNAs) were also identified, including 604 ribosomal RNAs (rRNAs), 749 transfer RNAs (tRNAs), 154 microRNA (miRNA) which belongs to a family of highly conserved post-transcriptional regulatory genes that play critical roles in various cellular and developmental processes, and 4032 small nuclear RNAs (snRNAs) that the length is about 150bp and is mainly found in the soluble part of the nuclear plasma, which is also associated with chromatin (**Supplementary Table S8**).

### 2.3 Gene family construction

**Figure 2:**
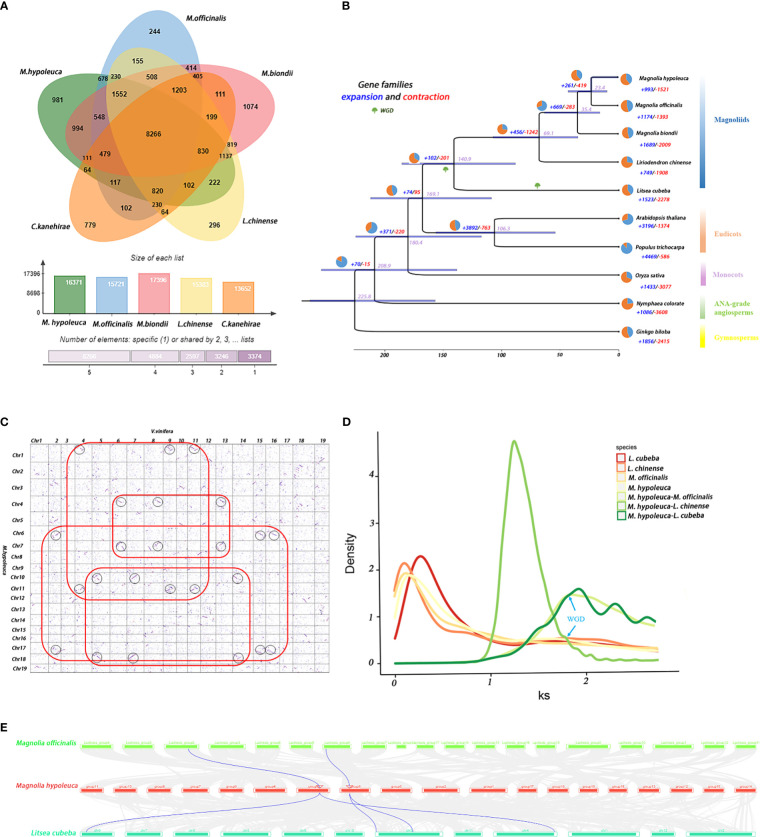
Evolution and comparative analysis of *M. hypoleuca*. **(A)** Venn-diagram and histogram of the number of common and species-specific gene families of *M. hypoleuca, M. officinalis*, *M. biondii*, *L. chinense, C. kanehirae*. **(B)** Phylogenetic tree based on 128 strictly single-copy homologous sequences identified by grouping homologous protein sequences of *M. hypoleuca* and the genomes of nine additional plant species. The purple numbers represent divergence time of each node (MYA), and the red and blue pie chart on each branch of the tree indicates the proportion of gene families experiencing contraction (red) or expansion (blue) events, and the red and blue numbers indicate the total number of expanded and contracted gene families. **(C)** Dot plots of orthologs showing X- (*V. vinifera*) and Y-axes (*M. hypoleuca*) are the numbers of cumulative genes on the respective 19 chromosomes for 1-1 chromosomal relationship. **(D)** The colored lines represent the Ks density distributions of paralogs and orthologs among four plant species (*M. hypoleuca, M. biondii*, *L. chinense* and *L. cubeba).* **(E)** Synteny patterns between genomic regions from *M. hypoleuca* to two regions in *L. cubeba* and to up to one region in *M. officinalis* (the blue wedges as a case highlighting a typical ancestral region.).

The single-copy orthologs from the predicted proteomes of *M. hypoleuca* with nine other sequenced species were combinated, including magnoliids, eudicots, monocots, ANA-grade angiosperms and Gymnosperms for the identification of homologous gene clusters. A total of 341,761 genes in 10 plant species were identified and consisting 310,964 homologous gene clusters, of which 24,403 clusters were common to all investigated species and represented ancestral gene families. Furthermore, 495 gene families containing 3,062 genes were found to be unique to *M. hypoleuca* among 30,952 gene families (**Figure 2A**; **Supplementary Figures S2, S3**, **Supplementary Table S9**). The Venn diagram shows that 8,266 gene families were commonly owned of magnoliids, including *M. hypoleuca, M. officinalis*, *M. biondii*, *L. chinense, C. kanehirae*.

In addition, 26,178 annotated functional genes were carried out from all predicted gene models through aligning against the contents of those functional database, such as NR, TrEMBL, KOG, Pfam, GO, and KEGG. According to GO analysis, 12,939 genes (54.66%) were annotated (**Supplementary Table S10**, **Supplementary Figure S4**) and the specific gene families were enriched in reproduction, cellular process, cell, cell part, binding, and nucleic acid binding. These specific genes of the *M. hypoleuca* related to secondary metabolite biosynthesis and plant-pathogen interactions lays a foundation for further research on in biological stress capacity mechanisms, provides a reference for breeding economically valuable, phylogenetic and ornamental tree species.

There were further KOG functional annotation studies showing that most of the genes in *M. hypoleuca* were functionally classified into multiple molecular families and annotated for secondary metabolites, transport and catabolism, which were enriched in posttranslational modification biosynthesis (1743 genes), transport and catabolism (924 genes), and signal transduction mechanisms (1488 genes) (**Supplementary Table S10**). KEGG pathway enrichment analysis revealed that *M. hypoleuca* genes were main performed to predict to the interaction of different metabolic pathways. The most highly represented category was carbon metabolism (324 genes), and the second is biosynthesis of amino acids (310 genes), followed by sucrose metabolism (249 genes) and phenylpropanoid biosynthesis (334 genes), etc. These results are consistent with the characteristics of *M. hypoleuca* containing high terpenoids and phenolic compounds (Miyazawa et al., 2015).

### 2.4 Phylogenomic placement of magnoliids sister to eudicots

In plant taxonomy, the phylogenetic relationships among monocots, eudicots and magnoliids have been disputed. To investigate the details in relation to the high-confidence phylogenetic tree of *M. hypoleuca* by great likelihood the analysis of tandem supermatrices and the divergence times were estimated based on 128 single-copy orthologs identified by grouping homologous protein sequences of *M. hypoleuca* and the genomes of nine additional flowering plant species have been fully sequenced, including 4 magnoliids (*M. officinalis, M. biondii, L. chinense, Litsea cubeba*), 1 Gymnosperms (*Ginkgo biloba*), 2 Eudicots (*Arabidopsis thaliana, Populus trichocarpa*), 1 Monocots (*O.sativa*), and 1 ANA-grade angiosperms (*Nymphaea colorate*). By using the program MCMCTree with calibrations, we further estimated the divergence times based on 128 strictly single-copy homologous sequences of *M. hypoleuca* and nine additional plant species. The phylogenetic tree shows *M. hypoleuca* clustered with four other magnoliids as expected, the divergence time between the *M. hypoleuca* and *M. officinalis* occurred about ~23.4 Mya, which shared a recent common ancestor with Liriodendron at ~69.1 Mya (**Figure 2B**).

It is consistent with previous studies that around 150 ~ 180 Mya, the magnoliids possibly have diverged from a branching lineage that includes core eudicots (Chen et al., 2019; Dong et al., 2021; Yin et al., 2021). However, the evolutionary distance scale between *M. hypoleuca* and *M. biondii* is shorter than between *M. officinalis* and *M. biondii*, which showing that *M. hypoleuca* and *M. biondii* has less genovariation compared with the latter. The relative evolution distance are in accord with biological characteristic among these three Magnoliaceae species, such as the fragrance. Both aggregation and linkage analyses yielded the same highly supported topology, indicating that magnolias forms sister groups with eudicotyledon after separation from monocotyledons. Together with previously research of the three genomes of Magnoliaceae (*M. officinalis*, *M. biondii* and *L. chinense*) (Dong et al., 2021; Yin et al., 2021), we concluded that the magnoliids and eudicots were placed as a sister to monocots. Thus, phylogenetic analysis of the combined *M. hypoleuca* genomes with other additional magnoliids species provides solid proof into the phylogenetic position and evolution.

**Figure 3:**
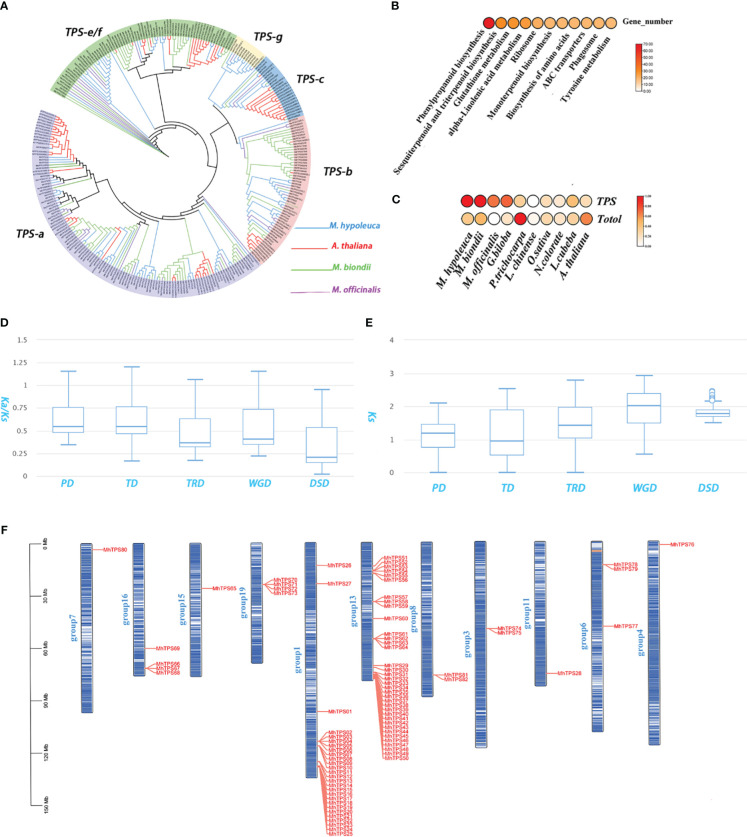
TPS gene family in *M. hypoleuca*. **(A)** Phylogeny of *TPS* genes from *M. hypoleuca* (82), *M. officinalis* (40), *M. biondii* (102), and *A*. *thaliana* (32). **(B)** *KEGG enrichment analyses of expanded genes*. **(C)** Distribution pattern of all expanded gene families and *TPS* genes comparison with other species. **(D, E)** Ka/Ks and Ks ratio distributions of gene pairs derived from different types of duplication. WGD, whole-genome duplication. **(F)** Chromosome localization of *M. hypoleuca TPS* genes.

A gene family is a group of genes that originates from the same ancestor and consists of two or more copies of a single gene through gene duplication, which have significant structural and functional similarities and encode similar protein products. Among them, gene family expansion or contraction plays an important role in the evolution of phenotypic diversity and fitness in plants (Chen et al., 2013), therefore, we examined whether each gene family undergoes dramatic expansion or contraction between those species (**Figure 3**; **Supplementary Tables S11, S12**). The results showed an expanded of 681 genes and a contracted of 2731 genes in the lineage number of this gene family. Further KEGG pathway enrichment analysis revealed that these expanded gene families were enriched in biosynthesis of secondary metabolites, pathogen-resistance, plant hormone signal transduction, including plant-pathogen interaction, terpene synthesis, phenylpropanoid synthesis pathways which were the major components of flower fragrance. This indicates that the expansion of gene family plays a significant role in the synthesis of fragrant substances of *M. hypoleuca*. In addition, we also conducted Gene Ontology (GO) enrichment analysis for the expanded gene families. The expanded gene families were assigned to functional groups in Gene Ontology (GO) enrichment analysis database, and the GO terms, such as ‘cell’, ‘cell part’ and ‘catalytic activity’ were detected hits, and large number of genes were hits fall into main functional category, namely, stimulus, metabolic process, single-organism.

### 2.5 Evolutionary history of Magnolia

Most plants have experienced ancient Whole-genome duplication (WGD) events or polyploidy, which is a massive chromosomal ploidy event that increases the dose of all genes in a species at once, resulting in a large number of chromosomal ploidy segments retained in the genome. In the above analyses, *M. hypoleuca*, *M. officinalis* and *M. biondii* were predicted that them were diverged from the most recent common ancestor, and most collinear blocks in the *M. hypoleuca* genome matched counterparts to the two genomes of the *M. officinalis* and *M. biondii*. The connectors of some putative homologous gene pairs in the chromosomes appear to cross, which may be caused by chromosomal inversion or recombination, which is consistent with the results of gene collinearity. There were 320 co-linear blocks containing 27,229 co-linear gene pairs retrieved between *M. hypoleuca* vs *M. officinalis* and 26,806 co-linear gene pairs from 410 co-linear blocks retrieved between *M. hypoleuca* vs *M. biondii* (**Supplementary Table S13**). To investigate sequence divergence and evolutionary relationships, dot plots of longer syntenic blocks of revealed that the nearly 1:1 orthology ratio (**Supplementary Figure S5**), and the chromosomes of *M. hypoleuca* and *M. officinalis* showed a good corresponding relationship, indicating that after the divergence of *M. hypoleuca* and *M. officinalis*, the chromosomes were conserved, with partial rearrangements. The dot plots of *M. hypoleuca* vs *M. biondii* also showed a good orthology ratio, but the linear comparison ratio slightly lower compared with *M. hypoleuca* vs *M. officinalis* (**Supplementary Figure S6**). Furthermore, there are 4517 co-linear gene pairs on 119 co-linear blocks in the *M. hypoleuca* genome, self-comparisons of the *M. hypoleuca* genome also indicated that there is another collinear block for a small fragment of chromosomes (**Supplementary Figure S7**).

It has been definitely proven that whole-genome triplication which mechanically originated in two consecutive WGD occurred in the *Vitis vinifera* (*V. vinifera)*. To confirm *M. hypoleuca* has undergone two rounds of WGD (including WGD of MRCA), we compared the syntenic depth ratio between *M. hypoleuca* genome to the genomes of *V. vinifera*. Similar to *M. biondii*, the approximately 2:3 orthology ratio scale between *M. hypoleuca* and *V. vinifera* by performing a comparative genomic analysis (**Figure 2C**). That confirmed after the most recent common ancestor of angiosperms (MRCA), Magnolia may also have experienced a WGD event. By calculating the Ks values of duplicate gene pairs, we observed a peak at Ks values of 1.78 indicating that Magnoliaceae likely underwent a WGD event and that the peak of ~0 values may be caused by tandem repeats. The peak value of orthologs between *M. hypoleuca* vs *M. officinalis* (Ks = 1.22) was lower than the value of Ks = 1.93 between *M. hypoleuca* vs *L. cubeba* (**Figure 2D**), implying that the speciation between *M. hypoleuca* and *M. officinalis* occurred later. The corresponding time points between *M. hypoleuca* vs *M. officinalis* and *M. hypoleuca* vs *L. cubeba* are ~ 22.3 and 140 Mya, and the WGD event shared by *M. hypoleuca*, *M. officinalis, L. chinense* and *L. cubeba* is about 1.78 ks. Thus, these indicated that all magnoliid species experienced a multiplication event about 115.32 Mya.

To confirm the WGD analyses that the *M. hypoleuca* and *M. officinalis* and Lauraceae shared this ancient whole genome duplication event, and *L. cubeba* have experienced an extra additional WGD event. Collinearity and synteny analysis between the *M. hypoleuca* vs *M. officinalis* and *M. hypoleuca* vs *L. cubeba* genome also provided clear structural evidence for two WGDs in Lauraceae with a 1:2 syntenic depth ratio in *M. hypoleuca* vs *L. cubeba* comparison, indicated two *L. cubeba* regions aligned to a single *M. hypoleuca* block (**Figure 2E**). Overall, syntenic evidence implied a single round of WGD in Magnoliaceae, and an additional round of WGD in Lauraceae.

### 2.6 The genes expansions, TD and PD may contribute to the fragrance accumulation

Flower fragrance is an important character of Magnolia, which plays a very significant effect in plant growth, development and evolution, and the main components of floral fragrances are a mixture of VOCs, which include terpenes, phenylpropanoids and fatty acid derivatives in Magnolia species. The KEGG results of further showed that the expanded genes sets were significantly enriched in the phenylpropanoid biosynthesis pathways, sesquiterpenoid and triterpenoid biosynthesis and monoterpenoid biosynthesis(**Figure 3B**). *TPS*s, the key enzyme for the synthesis of terpenes, was identified a total of 82 genes in the genome assembly of *M. hypoleuca* (**Supplementary Table S14**), which is significantly larger than that of *M. officinalis* with 40 genes. We find, quite interestingly, that which is significantly less than that of *M. biondii* (102 genes) which has more obvious scent. *TPS* family system is divided into 7 subfamilies, namely *TPS*-a, b, c, d, e/f, g and *TPS*-h. Among them, *TPS*-a, b and *TPS*-g exist in angiosperms and the *TPS*-a subfamily mainly synthesizes sesquiterpene synthase, and *TPS*-b and g mainly synthesize monoterpene synthase. *TPS*-c is closely related to *TPS*-e/f and mainly synthesizes diterpene synthase. *TPS*-c exists in gymnosperms and *TPS*-e/f exists in vascular plants. Phylogenetic analysis using all the *TPS* protein sequences from four species (*M. hypoleuca, M. officinalis*, *M. biondii, A. thaliana*) revealed that *TPS*s were clustered into five of six subfamilies: *TPS*-a (123 genes), *TPS*-b (56 genes), *TPS*-c (24 gene), *TPS*-e/f (12 genes), and *TPS*-g (73 genes). Most *MhTPS* cluster in the *TPS*-a and b subfamily group (**Figure 3A**), which are key enzymes for sesquiterpene and monoterpene synthases, respectively. *TPS* are the key enzymes responsible for biosynthesis of terpenoid compounds, which are also involved in significantly expanded genes in *M. hypoleuca and M. biondii* compared to *M. officinalis* (**Figure 3C**). Comparative genomics analysis showed that there were significantly expanded (68 genes) in a total of 82 *MhTPS*, in particular with the *TPS*-a subgroup (36 genes) and *TPS*-b subgroup (14 genes). The large expansion of *TPS*-a and b subfamily genes in *M. hypoleuca* may be the performance of improving abiotic stress in the long-term evolution process. These expanded *TPS*-a and b subfamily genes may contribute to the sesquiterpene and monoterpenes accumulation in *M. hypoleuca* (**Figure 3C**; **Supplementary Table S14**). The comparative analysis of *TPS*-a subfamily genes of *A. thaliana* and *M. officinalis* shows that the proportion of *TPS*-a and b subfamily genes is 2-3 times that of these two species, indicating that *TPS*-a has been greatly expanded in *M. hypoleuca*. In addition, a large number of *MhTPS* genes have been annotated as sesquiterpene and monoterpenes synthase genes, and seven *MhTPS* genes predicted in this study may

We identified 34 057 duplicated genes classified into five categories: 18 832 (55.29%) dispersed duplication (DSD) genes, 4571 (13.42%) WGD genes, 4788 (14.06%) transposed duplication (TRD) genes, 2576 (7.56%) tandem duplication (TD) genes and 3290 (9.39%) proximal duplication (PD) genes (**Supplementary Figure S8**). We compared the Ks, and Ka/Ks distributions among groups of duplicated genes with five different duplication modes. The Ka/Ks ratios among different modes of gene duplications showed a striking trend, with TD and PD gene pairs duplications having qualitatively higher than other three modes. suggesting might undergo selection for neofunctionalization or subfunctionalization. The Ks values of TD and PD genes were much smaller than those of other three modes (**Figures 3D, E**), suggesting that TD and PD genes formed recently and have been preserved have experienced more rapid sequence divergence than other gene classes (Qiao et al., 2019). *MhTPS* were allowed to be localized to chromosomes positions by the *M. hypoleuca* genome assembly, and the 82 *MhTPS* genes are not uniformly distributed in 11 chromosomes (**Figure 3F**). The expansion of the *MhTPS* family homologous genes was mainly caused by tandem repeation and WGD events that was similar to *M. officinalis* and *M. biondii*. There are two tandem repeats in the *TPS*-a subfamily gene family of *M. hypoleuca*, resulting in a total of nine genes (**Supplementary Figure S9**). Thus, the tandem duplications were largely responsible for TPS gene family expansion. KEGG functional enrichment analysis of the TD and PD gene sets demonstrates that they were mainly assigned in Phenylpropanoid biosynthesis, Sesquiterpenoid and triterpenoid biosynthesis, Monoterpenoid biosynthesis, Flavonoid biosynthesis, and Phenylpropanoid biosynthesis pathways which are critical for flower fragrance (**Supplementary Table S10**).

### 2.7 The genetic basis of cold and low temperature tolerance

**Figure 4:**
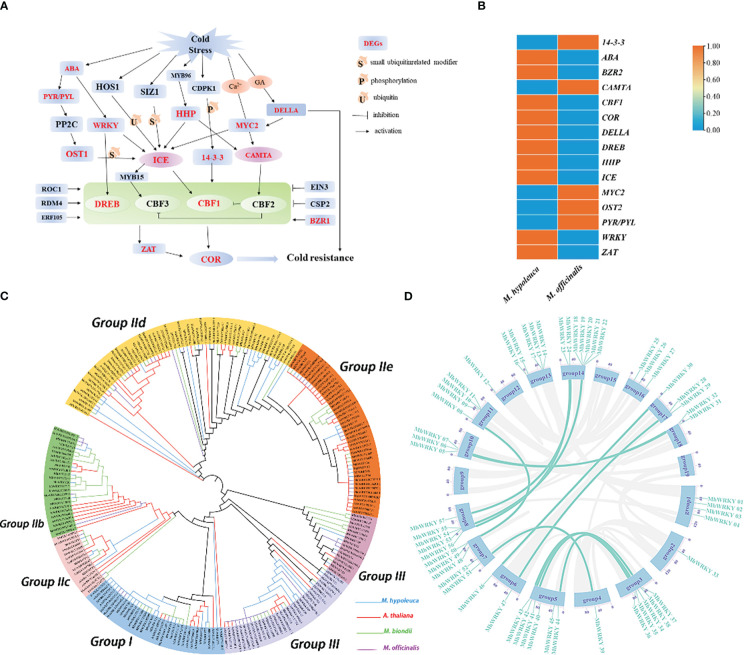
Cold resistance related genes in *M. hypoleuca*. **(A)** Schematic representation of key genes involved in central metabolic pathways of cold and low temperature tolerance in *M. hypoleuca*, and expression profiles of the relevant genes. **(B)** The cold resistance genes pathway DEGs identified in the *M. hypoleuca* and *M. officinalis* in this pathways. **(C)** Phylogeny analysis of *WRKY* genes from *M. hypoleuca* (55), *M. officinalis* (49), *M. biondii* (56), and *A*. *thaliana* (74). **(D)** Schematic representation of the *M. hypoleuca* chromosomes together with the positions of *MhWRKY* genes. The genes colored marked were generated by tandem duplicating events.

The cold tolerance characters are the another attractive features of *M. hypoleuca* which is also different from *M. officinalis*, and we analyzed the RNA-seq of tissue cultured plants of *M. hypoleuca* and *M. officinalis* to identify candidate genes responsible for the low temperature tolerance (0°C) of *M. hypoleuca.* A total of 2,118 differential expressed genes (DEGs) of *M. hypoleuca* cold stress tolerance were obtained compared to *M. officinalis* with a false discovery rate FDR<0.05 and log2 (change fold) (**Supplementary Table S15**). The results using GO analysis to understand the function of the DEGs mainly enriched in the ‘catalytic activity’ term (**Supplementary Figure S10**). KEGG pathway analysis of these genes revealed that many distinctive biological pathways were affected, such as plant-pathogen interaction, carbon metabolism, plant hormone signal transduction and Phenylpropanoid biosynthesis (**Supplementary Figure S11**). To explore cold stress-associated genes in *M. hypoleuca*, we also analyzed cold stress-associated gene families. 148 genes involved in cold signal transduction between *M. hypoleuca* and *M. officinalis* were identified (**Supplementary Table S16**). We surveyed the integrant expression profiles of genes belonging to ICE–CBF–COR cold responsive pathways (Wang et al., 2017) and researched substantial variations in expression between *M. hypoleuca* and *M. officinalis* (**Figure 4A**). Moreover, at least one DEG was identified for ten enzymes or transcription factors, and the up-regulation of *ABA, BZR2, CAMTA, CBF1, COR, DELLA, DREB, HHP, ICE, MYC2 OST1 PYR/PYL WRKY* and *ZAT*, illustrating that the ten of these enzymes in *M. hypoleuca* would be play a crucial part in enhancing cold tolerance (**Figure 4B**).

*WRKY* transcription factors, which are ubiquitous in plants, attributing to be the key factors in the response of plants to low temperature stress. A total of 55 *WRKY* genes were obtained in *M. hypoleuca*, which is approximately equal to *M. officinalis* with 49 genes. With the aid of the transcription data, a total of 58 *WRKY* genes were identified (**Supplementary Table S17**), the number of which is probably proportionable with assembly *M. hypoleuca* genome. Through phylogenetic analysis of the *MhWRKY* family, we found that the family members conform to the consistent classification of the *WRKY* family (**Figure 4C**). Except for a few *MhWRKY* proteins with zinc finger structure deletion, all other proteins have normal zinc finger structure. To some extent, the *MhWRKY* family is relatively conservative in evolution. According to the conserved domain sequence and zinc finger structure type of *WRKY* gene and the classification of Arabidopsis *WRKY* gene family, the *WRKY* gene family of *M. hypoleuca* is divided into three groups, including group I, group II and group III, which contain 11 genes, 36 genes and 8 genes respectively. The *WRKY* gene family group II of *M. hypoleuca* can be further subdivided into 5 subfamilies, IIa-e, containing 4, 5, 3, 16 and 8 genes respectively. *MhWRKY* genes located in Chr3, Chr4, Chr 6, Chr8, and Chr10 are tandem duplicating genes, and a large number of fragmented duplicating genes exist on the other 11 chromosomes (**Figure 4D**), and the proportion of tandem gene pairs is 48.28% in *MhWRKY* family (**Supplementary Figure S8**). In order to understand whether *MhWRKY* was affected by natural selection in the evolutionary process, Ka/Ks analysis is conducted on tandem duplicating genes and fragment duplicating genes. The results showed that the majority of duplicated *MhWRKY* genes evolve under purifying selection to eliminate harmful mutation sites (Ka/Ks <1). The stronger selective pressure drived the evolution of tandem and proximal duplicates toward specific biological functions, and we performed GO enrichment analysis to investigate the functional roles of TD and PD genes in the model plant *M. hypoleuca*, given its high-quality genome annotation and extensive functional analysis. Tandem and proximal duplicates exhibited divergent functional roles although they shared several enriched GO terms involved in metabolic process, catalytic activity, binding, single-organism process and cellular process, which are critical for plant self-defense and adaptation (**Supplementary Table S10**, **Supplementary Figure S12**). In additition, we found that two members (*MhWRKY*2 and *MhWRKY* 28), have eight cis-acting elements related to plant growth and development and stress tolerance through promoter analysis, which indicates that these two members of *MhWRKY* are likely to have a very important relationship with plant development and stress resistance (**Supplementary Table S18**).

## 3 Discussion

Considering the medicinal, biological and phylogenetic importance of Magnoliaceae, this high-quality assembled genome provides an important reference for evolutionary and functional studies of *M. hypoleuca*, especially related to studies of cold resistance and floral fragrance. We obtained a high quality and chromosome-scale genome of *M. hypoleuca* based on the combination of high coverage Illumina sequencing, Oxford Nanopore technology of long-read sequences and Hi-C technology of highly accurate reads. This powerful assembly allowed us to map the genome of *M. hypoleuca* in detail, including its numerous repetitive elements, accounting for 64.54% of the assembly. The intense fragrance and cold tolerance characters are the attractive features of *M. hypoleuca* which is also different from *M. officinalis*. However, there were higher coverage and more protein-coding gene sequences in this genome with highly homologous comparing with the *M. officinalis* genome. The genome integrity assessment based on BUSCO database shows that the genome assembly of *M. hypoleuca* and *M. officinalis* can cover 95.5% and 93.05% of the conserved plant gene sets, respectively, indicating that the genome assembly of *M. hypoleuca* may be more complete than *M. officinalis*. This reference genome provides genetic profiles, repetitive elements and genomic architectures of DNA on 19 chromosomes, and our genomic data provide precious molecular and applied genetic resources for the evolutionary and comparative genomic studies of Magnolia and its related species.

The phylogenetic relationships among basal taxa of angiosperms have become increasingly unambiguous as the genome sequences of early evolved flowering plant species are increasingly reported. In addition, the phylogenetic analysis employing amino acid sequences and nucleotide sequences showed that the clade genus of Magnolia and eudicots was sister to the genus Monocotyledon after the common ancestor of Magnolia and Eugenia diverged from Monocotyledon, *which agrees with a previous study using the C. kanehirae, Litsea cubeba and C. salicifolius genome* (Chen Y. C. et al., 2020; Lv et al., 2020; Shang et al., 2020), and this is inconsistent with the phylogenetic analytical view observed in previous studies of Magnolias (Lv et al., 2020; Dong et al., 2021; Yin et al., 2021). The two rounds of WGD events indicate that be similar to Liriodendron, the Magnoliales experienced the common WGD event by the analysis of ortholog divergences, Ks plots, phylogenomic analyses, and synteny, which is consistent with the conclusion of the previous studies of Magnolias.

At present, *M. hypoleuca* is only native of Japan, other places such as China, Korea are introduced, which can survive the winter safely at -25 °C, and *M. officinalis* in southern China will suffer frost damage in the area north of the Yellow River. *M. hypoleuca* is one of the most fragrant flowering trees and can be smelled tens of meters away, but *M. hypoleuca* is a light fragrance, which can only be smelled when you are close. The differentiation of *M. hypoleuca* and *M. officinalis* is Oligocene-Miocene transition (OMT, 25 ~ 22 MA) which is a period of intense geological and historical events caused by the collision of the Indian plate and Eurasia (Zhao and Li, 2019). The rapid uplift of the Qinghai-Tibet Plateau and Himalayas, the rapid lateral sliding of the Indochina Peninsula and the strengthening of the monsoon occurred at this stage (Liebrand et al., 2017). The uplift of the Qinghai-Tibet Plateau and the Himalayas is one of the most significant meteorological and geological events in the Cenozoic era, which not only affects the structural pattern of Eurasia, but also has a profound impact on the global climate change since the Cenozoic era. The Japanese Archipelago originated in the Miocene as an independent part of land masses in eastern and western Japan (Ninomiya et al., 2014). These ecological events contributed to the evolution of plants from the mainland and their later divergence as characteristic lineages (i.e., new species), greatly contributing to the high floristic diversity of Japan. The climate change of OMT is the main reason for the divergence of *M. hypoleuca* and *M. officinalis*. The divergence of *M. hypoleuca* was followed by the division of Japanese islands. Its floral fragrance and cold resistance are the result of adapting to the changing environment during its long years in the Japanese archipelago. In conclusion, these persistent genetic variants in *M. hypoleuca* associated with key biological functional traits may contribute to adaptation to possible future climate change.

Gene family expansion and specificity gene analysis showed that the *M. hypoleuca* genome comprises more floral scent-related expansion genes compared to *M. officinalis*, consistent with hy containing a more intense floral scent. There are remarkable discrepancies in the populations of *TPS* gene families in individual plants. In angiosperms, the number of *TPS* genes is about 40-152 (Chen et al., 2011). By gene family analysis, we detected a total of 82 putative *TPS* genes in *M. hypoleuca* genome, higher than those identified in *M. officinalis* (40), *A. thaliana* (64). Comparison of the gene tree topology of four angiosperm *TPS* proteins showed that *TPS* genes were expansed in *M. hypoleuca*, especially for *TPS*-a and *TPS*-b subfamily members. Previous studies have shown that 32 functional *TPS* genes have been found in Arabidopsis (Irmisch et al., 2014). In this study, 48 functional *MhTPS* genes encoding different terpene synthetases were predicted. Neroli tertiary alcohol, linalool and other aromatic substances with magnolias are the main components of aroma, and a large number of *MhTPS* genes have been annotated as sesquiterpene synthase genes. which are the same as the annotated functions of *TPS*-g gene family in tomato and poplar (Zhou et al., 2020). The *MhTPS* genes predicted in this study are based on the analysis of homologous genes with known functions of plant terpenoid synthetases, which have high sequence alignment span and similarity. However, the reciprocal interactions between these genes and how they systematically modulate the composition and expansion of flower fragrance still need to be pursued further.

We surveyed the integrant expression profiles of genes belonging to ICE–CBF–COR cold responsive pathways (Wang et al., 2017), we researched substantial variations in expression between *M. hypoleuca* and *M. officinalis* and ten enzymes of these DEGs in *M. hypoleuca* would be play a crucial part in enhancing cold tolerance. The *WRKY* family is one of the largest families of transcription factors that are not only plant-specific but also implicated in biological processes such as plant growth and development, biotic and abiotic stress tolerance (Jiang et al., 2017). Through the analysis of conserved motifs and gene structure, we found a conserved domain motif which is likely to be the core conserved domain of the *MhWRKY* family from all members of the *MhWRKY* family. In the transcriptome data, these observations above suggested that adaptive evolution with abundant cold and low temperature tolerance properties in *M. hypoleuca* can be induced by selective expression of the corresponding enzyme activities. *MhWRKY*2 and *MhWRKY*28 are also up-regulated in *M. officinalis*, that are still worthy of further study. Among them, 25 *MhWRKY* members have low temperature responsiveness, and all *MhWRKY* members have ABA response elements, and ABA usually acts as an anti-stress hormone in plants, which also reflects that the *MhWRKY* family are important for the response mechanism of plants cold and low temperature tolerance (Zhou et al., 2015). There are studies that proved that *WRKY*22 gene members were implicated in drought stress, whereas *WRKY*28 and *WRKY*33 was correlated with the cold stress (Balti et al., 2020). *ATWRKY*22 promotes aphid predisposition in Arabidopsis and regulates salicylic and jasmonic acid signaling. Some studies have shown that *WRKY*33 is critical to the tolerance of plants to abiotic stress. At 45°C, the *ATWRKY*33 mutant is highly sensitive to heat stress (Zhou et al., 2015). Yuan found that when *Brassica Juncea* was treated with hormone (ABA), cold injury (4°C) and salt injury, the expression abundance of *BJWRKY*33 gene was increased, indicating that BJ*WRKY*33 gene may be important for these three kinds of stress (Yuan et al., 2019). When *M. hypoleuca* and *M. officinalis* was subjected to cold stress, the expression of *MhWRKY*33 was significantly up-regulated. It is speculated that *MhWRKY*33 may participate in cold stress. In general, the *WRKY* gene family in *M. hypoleuca* may be able to increase the tolerance of low temperature stress, and *WRKY*33, *WRKY*22 and *WRKY*28 may have a potential role in improving the cold resistance of *M. hypoleuca*.

Taken together, the results allow us to deeply investigate the evolution and genetic diversity of *M. hypoleuca*, as well as the fragrance and cold tolerance they produce, and provide a more compelling basis for a better understanding of the evolution and diversity of Magnoliales.

## 4 Conclusion

We report a high-quality and chromosome-scale genome assembly of *M. hypoleuca*, the special species in the genus Magnoliaceae. The genome of *M. hypoleuca* has a contig N50 of 1.71 Mb and is distributed into 19 chromosomes. The Magnoliaceae, including *M. hypoleuca, M. officinalis, M. biondii* and *L. chinense*, diversified for approximately 140 mya with Lauraceae family, represented by *L. cubeba*. The climate change of OMT is the main reason for the divergence of *M. hypoleuca* and *M. officinalis*, which was along with the division of Japanese islands. In addition, We found that *TPS* genes, particularly those from the *TPS*-a and *TPS*-b group, significantly expanded and thus are key potential targets for flower fragrance. Approximately two-thirds of the *WRKY* genes were upregulated under cold in *M. hypoleuca*, indicating their importance in low temperature adaptation. Overall, our study provides support for further studies of floral evolution *M. hypoleuca* evolution, flower fragrance and low temperature adaptation.

## 5 Materials and methods

### 5.1 Plant materials, DNA and RNA extraction and sequencing

We collected fresh leaves material from a fully mature tree growing for DNA extraction in Dandong, Liaoning Province, China. and the transcriptome material of buds at same developmental stages were collected from the six individual trees planted (*M. hypoleuca* and *M. officinalis*) with three biological replicates in the Dandong, Liaoning and Mianyang Sichuan. The low temperature treatment were conducted with *in vitro* plants of the same age, these plants were placed under 0°C conditions, and buds were collected at a week during the cold treatment. The nucleic acid was extracted from those buds material of *M. hypoleuca* using DNA secure Plant Kit (TIANGEN) and Total RNA Kit (TIANGEN).

Store all materials in -80°C refrigerator for Oxford Nanopore Technologies (ONT) sequencing after the quality and quantity of the DNA samples were evaluated, the DNA libraries with insert size of 20- 40kb were sequenced on PromethION platform, and a total of 306.87 Gb raw data were obtained. A total of 288 Gb of clean data was retained using software for adapter trimming and quality filtering. Plants from the same genetic background as Oxford nanopore sequencing were used for Hi-C library construction and sequencing. Young buds of *M. hypoleuca* were collected using biotin-labeled DNA fragments enriched with beads for DNA sequencing library construction. The library was then sequenced on the Illumina HiSeq 4000 platform in a paired-end 150 bp length pattern. A total of 88 Gb (~52×genome coverage) of clean data was generated after quality control using fastp (Chen et al., 2018) (version 0.12.6) with default parameters. Hi-C data were evaluated for quality using HiC-Pro software.

RNA samples with integrity values close to 10 were selected for cDNA library construction and subsequent sequencing by a series of quality control methods such as NanoDrop™ One UV-Vis spectrophotometer and a Qubit™4.0 Fluorometer. The cDNA library was constructed to sequence using the Illumina NovaSeq 6000 platform followed by paired-end (150 bp) sequencing.

### 5.2 Genome assembly and chromosome construction

Before assembly, raw ONT reads were self-calibrated with NextDenovo (https://github.com/Nextomics/NextDenovo), two software programs, Canu v1.8 (Koren et al., 2017) and WTDBG (Ruan and Li, 2020) were used for calibration assembly. The assembly genome of *M. hypoleuca* were optimally performed by two methodologies by using the consolidation ideation of Quickmerge (Chakraborty et al., 2016). Finally, second round of polishing for of error correction with short paired-end reads generated, the following parameters and diploid with Pilon (Walker et al., 2014) was applied.

In order to immobilize the contigs to the 19 chromosomes (Zhang et al., 2019), the Hi-C library was constructed in the Illumina HiSeq 4000 platform. After assessment of the quality of the Hi-C clean data using the HiC-Pro v2.7.1 (Servant et al., 2015), the clean Hi-C data were aligned to initially genomically assembled fragment using BWA v0.78 (Li and Durbin, 2010). These corrected genomic clusters were then clustered, sorted, and oriented to genomes with chromosomes by ALLHIC. The contigs genome is indexed and the HIC sequences are back-pasted, and the resulting SAM files are pre-processed to remove redundant and low-quality signals to improve processing efficiency. The filtering criteria of ‘filterBAM_forHiC.pl’ is that the matching quality is higher than 30%(MQ), only unique matches are retained, the editing distance is lower than 5, the error match is lower than 4, and there cannot be more than 2 gaps. And build Allele.ctg.table for filtering the false signals that will appear between different sets of genomes. HiC signal will group different contigs, the number of groups is controlled by -k, assign unanchored contigs to existing groups, optimize the ordering and orientation of contigs in each group, convert the tour format to fasta format and then build Chromatin interaction matrix, evaluate the results according to the heat map.

### 5.3 Genome assembly quality evaluation

The contiguity, correctness and integrity of the *M. hypoleuca* genome assembly were evaluated from four perspectives. First, we evaluated the coverage degree of the clean raw reads from transcriptomes and genome obtained from the assembly by TopHat2 (Kim et al., 2013) and BWA-MEM (Li and Durbin, 2010) with default parameters, respectively. In general, 50% of contigs covering more than 95% of the genes and 85% of contigs covering more than 90% of the genes are considered as more complete assemblies. There are always conserved sequences between similar species, and BUSCO uses these conserved sequences to compare with the assembly results to identify whether the assembly results contain these sequences, single, multiple or partial or not, etc. to give the results. We researched the BUSCO (Simao et al., 2015) (v 5.0) genes in the final assembly. Finally, we used the LAI (Ou et al., 2018) which is ratio of complete LTR inversion motif sequence to total LTR sequence length to infer the assembly continuity.

### 5.4 Repeat annotations

Transposable element (TEs) also known as jumping genes, are sequences of DNA that can change their position and were identified using a combination of evidence-based search and abinitio prediction approaches. For evidence-based search, *M. hypoleuca* genome was searched against the Repbase database (Bao et al., 2015) (v.20.05) using RepeatMasker (Tarailo-Graovac and Chen, 2009) (v.4.1.27) (http://www.repeatmasker.org/) which is a common tool for genomic repeat sequence detection with default parameters. It generally relies on an existing repetitive sequence reference library, Repbase, for homology prediction. The current whole genome sequence is used to train the repetitive sequence set to construct a local repeat library, and then the repetitive sequences are annotated by RepeatMasker. Among them, RepeatModeler, a companion to RepeatMasker, can be implemented. Then, LTR_harvest and LTR_FINDER are used to find potential LTR sequences in *M. hypoleuca*, respectively, and LTR_retriever (Xu and Wang, 2007; Ou and Jiang, 2018) (v.1.8.0) is used to merge the results afterwards and build an LTR library with default parameters, then GeMoMa (Keilwagen et al., 2016) v1.3.1 was employed for the homology-based prediction. In fact, since substitutions between pyrimidines and purines are more difficult than substitutions between homologous bases, the Kimura 2-parameter model is used when considering transitions and transversions separately. RepeatProtein Mask was performed to detect TEs in the *M. hypoleuca* and *M. officinalis* genome by comparing the TE protein database. Results from these two runs of RepeatMasker were merged.

### 5.5 Gene predictions

After performing the necessary genomic repeat sequence masking for *de novo* gene searching using Genscan v3.1 (Burge and Karlin, 1997), Augustus v2.4 (Stanke et al., 2006), GlimmerHMM v3.0.4 (Stanke et al., 2006), GeneID v1.4 (Parra et al., 2000), and SNAP (Johnson et al., 2008) software. Prediction of genes in the genome (exon and intron regions) based on protein sequences using exonerate software (Slater and Birney, 2005) to achieve evidence for gene structure. Based on HMM (Hidden Markov Chain) and Bayesian theory for ab initio prediction, AUGUSTUS software (Stanke et al., 2006) is used to train the software with annotation information of existing species thickets to infer the possible structures in a gene sequence from the training results, and also to integrate EST, cDNA, and RNA-seq data as *a priori* models for prediction. A gene predicted to be positively transcribed is programmed to extend 12,000bp from the original comparison, and select the predicted result when it encounters a place that can be filled in PASA (Program to Assemble Spliced Alignments, v2.0.1) is a eukaryotic genome annotation tool that automatically generates gene structure annotations using splice comparisons of transcript sequences and maintains as much consistency as possible with experimental data. PASA is also able to identify almost all variable splices and predict the open reading frames (ORFs) in the transcripts. Finally, all the predictions were combined into consensus gene models using EVM (Haas et al., 2008).

### 5.6 Functional annotation of protein-coding genes

Whole-genome sequencing will generate a large amount protein-coding genes of *M. hypoleuca*, and previously it is common to use a comparison method to annotate the predicted coding gene with function, and obtain the functional information of the gene by protein comparison with various functional databases [NR (Marchler-Bauer et al., 2011)], Swiss-Prot protein (Swiss-Prot) (Boeckmann et al., 2003), Gene Ontology (GO) (Harris et al., 2004), Clusters of Orthologous Group (COG) (Tatusov et al., 1997), eukaryotic Orthologous Groups (KOG) (Tatusov et al., 2001), eggNOG4.5, Kyoto Encyclopaedia of Genes and Genomes (KEGG) (Kanehisa et al., 2017) and other databases). Among them, GO and KEGG databases occupy an important position in the study of gene function and metabolic pathways, respectively. The information obtained through genome annotation can be further used for subsequent comparative genomic analysis. Rfam is a database used to identify non-coding RNAs and is often used to annotate new nucleic acid sequences or genomic sequences. Call blast to compare the query sequence to Rfam.fasta’s blast database to find similar sequences of ncRNA with an E-value threshold of 0.01, and then call cmsearch with the blast result to verify using Rfam.cm.

### 5.7 Gene family and phylogenomic construction

Protein and nucleotide sequences from *M. hypoleuca* and four other magnoliales (*M. officinalis*, *M. biondii* and *L. chinense*, *L.cubeba*) were used to construct gene families. OrthoFinder infers a gene tree based on an all-versus-all BLASTP (Chan et al., 2021) for all orthologous groups by finding the direct and orthologous groups, and infers a rooted species tree for the species analyzed with an e-value cut off of 1e^−5^. Potential gene pathways were identified by gene targeting to the KEGG database (Kanehisa and Goto, 2000), and Gene Ontology (GO) terms were extracted from the corresponding interproscan or Pfam results.

To understand the relationships between the *M. hypoleuca* gene families and those of other plant species and the phylogenetic placements of magnoliids among angiosperms, We analyzed the protein-coding genes of different species during the phylogeny of 10 diverse taxonomic groups of plants from 128 strictly single-copy nuclear genes, including five magnoliids (*M. hypoleuca, M. officinalis, M. biondii, L. chinense, L.cubeba*), two Eudicots (*A. thaliana, P.trichocarpa*), one Monocots (*O.sativa*), one ANA-grade angiosperms (*N.colorate*) and one Gymnosperms (*G.biloba*). Orthologous gene groups of *M. hypoleuca* and nine other species were identified by running OrthoMCL (Li et al., 2003) program (http://orthomcl.org/orthomcl/).

### 5.8 Analysis of genome synteny and WGD

Ka/Ks represents the ratio between the non-synonymous substitution rate (Ka) and the synonymous substitution rate (Ks) of two protein-coding genes. This ratio can determine whether there is a selection pressure acting on this protein-coding gene. The Ka, Ks, and Ka/Ks values were estimated for *M. hypoleuca, M. officinalis, L. chinense* and *M. biondii* by hybridization and monophyletic species analysis based on the YN model in KaKs_Calculator2 (Wang et al., 2010). The Ks values also have some unavoidable limitations, for example, it is difficult to apply them to the identification of older genome doubling events because the Ks values between putative homologous gene pairs change over time, and for old genome doubling events. The Ks values of the putative homologous gene pairs generated vary widely or slightly, which is finally reflected in the distribution of the Ks values to find some Ks with large variance. This makes it difficult to project whether there is an obvious Ks peak. In addition, as synonymous substitutions tend to saturate with time, this can lead to bias in the calculation of Ks values, causing difficulties for finding ancient genome doubling events. Generally speaking that each peak corresponds to a species segregation or genome-wide doubling event, and the equation T = Ks/2r (r = 3.21 × 10^-9^) was used to calculate WGDs events and orthogonal divergence.

BLASTP (Chan et al., 2021) was used to match each genome pair by searching for putative homologous and homozygous genes within and between genomes by DIAMOND (Buchfink et al., 2015). We performed a modified MCScan (Wang et al., 2012) algorithm to analyze covariance syntenic blocks within *M. hypoleuca*, *M. officinalis* and *M. biondii* using MCScanX. It uses two species protein blastp comparison results, combined with the position of these protein genes in the genome, to obtain the covariance blocks in the genomes of the two species blocks. Homologous gene identification using orthoMCL, followed by selection of direct homologs for species tree construction, and finally gene family expansion and contraction analysis of the clustering results using CAFÉ (De Bie et al., 2006) (P < 0.01). The species tree was then used as an input to estimate divergence time in the MCMCTree program of the PAML package (Yang, 2007) based on the multiple fossil times from TimeTree(http://www.timetree.org/). CAFE applies a random birth-death model to simulate gene family gain or loss during a phylogeny. To infer the phylogenetic process, the gene family size transfer rate from parent to child nodes can be calculated, and the gene family size of ancestral species can also be inferred. The expansion and contraction of gene families were inferred based on the chronogram of the above-mentioned ten plant species.

### 5.9 Identification of TPS genes fragrance accumulation

To identify the pattern of *M. hypoleuca* genome-wide duplications, we divided duplicated genes into five categories, including WGD, TD (two adjacent duplicates), PD (duplicate genes separated by less than 10 genes on the same chromosome), TRD (duplicate gene composed of an ancestor and a new locus), and DSD (duplicate genes that are neither adjacent nor collinear) gene, using DupGen_Finder (Qiao et al., 2019; Zwaenepoel and Van de Peer, 2019) with the default parameters. The Ka, Ks, and Ka/Ks values were estimated for duplicated gene pairs with consistent calculation method mentioned in 5.8. HMMER (Eddy, 2011) 3.2.1 software HMM Seed profile (PF01397 and PF03936) containing the *TPS* structural domain were downloaded from the Pfam (http://pfam.xfam.org/) database and searched for the *M. hypoleuca TPS* gene family using HMMER3.1 software. The protein sequences collected were compared with the *M. hypoleuca* and *A. thaliana* protein sequences obtained from the Arabidopsis information resource (TAIR) (https://www.arabidopsis.org/) by BLASTP (Chan et al., 2021) methods, and the genes with high confidence were screened out according to the threshold condition of E-value <1×10-10. The obtained protein sequences were further verified by the NCBI CDD (https://www.ncbi.nlm.nih.gov/cdd/) database and SMART (Letunic et al., 2015) and after manual confirmation, the non-full-length sequences were removed and the candidate sequences were obtained and removed, the candidate sequences with full-length open reading frames (ORFs) were obtained for the *TPS* family members. The final 82 Mh*TPS* family members were obtained. *TPS* gene family sequences were mapped by MAFFT (Katoh and Standley, 2013) and phylogenetic trees were constructed by PhyML (Guindon et al., 2010). The tree was constructed by the maximum likelihood method (Jones et al., 1992) with the JTT matrix model and the bootstrap method with 1000 replicate the phylogenetic tests. The obtained phylogenetic trees were consequently visually analyzed using the online website iTOL (https://itol.embl.de). Synteny and collinearity syntenic blocks between these Mh*TPS* family members genes were analyzed using MCScanX (Wang et al., 2012). The structure and chromosome distribution of the Mh*TPS* gene was described using TBtools (Chen C. et al., 2020) based on the chromosome localization in the gff file of the genome structure annotation file.

### 5.10 Analysis of genes involved in the formation of strong cold resistance

Transcriptome data between *M. hypoleuca* and *M. officinalis* were obtained, and Salmon v1.3.0 (Patro et al., 2017) was used to quantify gene expression, with the default settings. The sequences involved in the central metabolic pathways of cold and low temperature tolerance and regulatory mechanisms in *A. thaliana*, were used to against the protein database of *M. hypoleuca*. We then internally check the alignments manually and eliminate any apparently discrepancies in the sequences. The expression level of *M. hypoleuca* and *M. officinalis* related genes was drawn into a heat map with the TBtools in cold and low temperature tolerance.

The gene sequence of *WRKY* in *M. hypoleuca* was obtained using the same method as *TPS* gene identification, and the *WRKY* protein sequence of *M. hypoleuca, M. officinalis, M. biondii* and *A. thaliana* was subjected to multiple sequence alignment using MEGA 7 software (Kumar et al., 2018) with default parameters, and then a phylogenetic tree was constructed by the neighbor-joining method with bootstraps set to 1,000 repetitions and the rest parameters as default. The conserved structural domains of *WRKY* family protein sequences were predicted using the online software MEME (http://meme-suite.org/tools/meme), and the conserved motif results were displayed using TBtools software. GSDS2.0 online website (http://gsds.gao-lab.org/) was used to map the gene structures of 55 *WRKY* gene family members of Japanese thicket, and their exon number/coding regions (CDS) were analyzed; the *WRKY* phylogenetic tree, conserved structural domains and gene structure results were visualized in evolutionary tree order using TBtools software.

Cis-actingelement is a sequence that is present in the paralogous sequence of a gene and can affect gene expression. They include promoters, enhancers, regulatory sequences and inducible elements, which are involved in the regulation of gene expression. The 2000-bp region up-stream obtained ‘Gff3 Sequence Extraction’ using TBtools software of *MhWRKY* genes was defined to extract of promoter sequences of the target gene set, and submitted to PlantCARE website (Lescot et al., 2002) and PLACE (Higo et al., 1999) databases for homeopathic component prediction.

## Data availability statement

The data presented in the study are deposited in the China National GeneBank DataBase (CNGBdb) repository, accession number CNP0003464 (https://db.cngb.org/search/project/CNP0003464/).

## Author contributions

JG XQ, and LW designed the project, LZ, FH, XQ, and LYZ collected samples and performed DNA sequencing. LYZ, YY, and WY assembled the genome, LZ, FH, and LZ performed RNA-seq. LZ, YY, WY, and CP wrote and revised the manuscript. All authors contributed to the article and approved the submitted version.
