# Supplementary material for: The genome of Magnolia hypoleuca provides a new insight into cold tolerance and the evolutionary position of magnoliids
Source: Front Plant Sci. 2023 Feb 10;14:1108701. doi: 10.3389/fpls.2023.1108701 (PMC9950645; doi:10.3389/fpls.2023.1108701)
Supplement: Supplementary file 1 [file DataSheet_1.docx]

Supplementary Material

# Supplementary Data

**Supplementary Table S1**. Summary of sequencing data of *M.hypoleuca*.

**Supplementary Table S2.** Statistic of *M.hypoleuca* genome assembly.

**Supplementary Table S3.** Chromosomes length of *M.hypoleuca* using Hi-C reads.

**Supplementary Table S4.** Assembly results of M.hypoleuca using Hi-C.

**Supplementary Table S5.** Validation of genome assembly using BUSCO.

**Supplementary Table S6.** Annotation of repeat sequences in the *M.hypoleuca* genome.

**Supplementary Table S7.** Statistics of predicted protein-coding genes in *M.hypoleuca* genome.e.

**Supplementary Table S8**. Annotation of conserved non-coding RNA genes in the *M.hypoleuca* genome.

**Supplementary Table S9**. *M.hypoleuca*-specific gene families.

**Supplementary Table S10**. Functional annotation of the *M. hypoleuca* protein-coding genes and duplicated genes.

**Supplementary Table S11**. The annotation of expanded genes in *M. hypoleuca* genome.

**Supplementary Table S12.** The annotation of contracted genes in *M.hypoleuca* genome.

**Supplementary Table S13**. Tandem duplicated gene clusters in *M.hypoleuca* genome.

**Supplementary Table S14.** Table S14. Terpene synthase family in *M. hypoleuca.*

**Supplementary Table S15**. Differently expressed genes between *M.hypoleuca* and *M.officinalis*.

**Supplementary Table S16.** Table S16. Differently expressed genes of cold signal transduction between *M. hypoleuca* and *M. officinalis.*

**Supplementary Table S17.** WRKY family in *M. hypoleuca.*

**Supplementary Table S18**. Promoter prediction of *M.hypoleuca.*

**Supplementary Fig. S1**. Automatic Hi-C map of the *M.hypoleuca* genome showing genome-wide all-by-all interactions. The map shows a high resolution of individual chromosomes that are scaffolded and assembled independently. The heat map colors ranging from light yellow to dark red indicate the frequency of Hi-C interaction links from low to high (0–10).

**Supplementary Fig. S2**. The distribution of single-copy, multiple-copy, unique, and other orthologs in the 10 plant species.

**Supplementary Fig. S3**. Sharing of gene families by *M.hypoleuca* and four other species. Cluster count is the number of clusters shared between species.

**Supplementary Fig. S4**. GO and KOG [function annotation](javascript:;) of protein sequence in *M.hypoleuca*.

**Supplementary Fig 5**. Comparison of [*M.hypoleuca*](https://kns.cnki.net/KNS8/Detail/RedirectScholar?flag=TitleLink&tablename=GARJ2017&filename=SJES1FE3D008D08A03BAE10AD66EDECA67D6) *and M.officinalis* genomes. Dot plots of orthologs showing a 1–1 chromosomal relationship between the [*M.hypoleuca*](https://kns.cnki.net/KNS8/Detail/RedirectScholar?flag=TitleLink&tablename=GARJ2017&filename=SJES1FE3D008D08A03BAE10AD66EDECA67D6) *and M.officinalis* genome.

**Supplementary Fig 6**. Comparison of [*M.hypoleuca*](https://kns.cnki.net/KNS8/Detail/RedirectScholar?flag=TitleLink&tablename=GARJ2017&filename=SJES1FE3D008D08A03BAE10AD66EDECA67D6) *and M.biondii* genomes. Dot plots of orthologs showing a 1–1 chromosomal relationship between the [*M.hypoleuca*](https://kns.cnki.net/KNS8/Detail/RedirectScholar?flag=TitleLink&tablename=GARJ2017&filename=SJES1FE3D008D08A03BAE10AD66EDECA67D6) *and M.biondii* genome.

**Supplementary Fig 7**. Self-collinearity map of [*M.hypoleuca*](https://kns.cnki.net/KNS8/Detail/RedirectScholar?flag=TitleLink&tablename=GARJ2017&filename=SJES1FE3D008D08A03BAE10AD66EDECA67D6). The values on the X- and Y-axes are the numbers of cumulative genes on the 19 chromosomes.

**Supplementary Fig 8**. Categories and proportions of different types of duplicated genes in *M. hypoleuca* and *MhTPS, MhWRKY*. TD, tandem duplication; PD, proximal duplication; TRD, transposed duplication; DSD,dispersed duplication.

**Supplementary Fig 9.** Intergenomic synteny blocks among 9 *TPS* genes of [*M.hypoleuca*](https://kns.cnki.net/KNS8/Detail/RedirectScholar?flag=TitleLink&tablename=GARJ2017&filename=SJES1FE3D008D08A03BAE10AD66EDECA67D6)*.*

**Supplementary Fig 10**. All Unigene of [*M.hypoleuca*](https://kns.cnki.net/KNS8/Detail/RedirectScholar?flag=TitleLink&tablename=GARJ2017&filename=SJES1FE3D008D08A03BAE10AD66EDECA67D6) and *M.officinalis* and GO classification of DEG.

**Supplementary Fig 11**. KEGG enrichment and classification of differentially expressed genes in [*M.hypoleuca*](https://kns.cnki.net/KNS8/Detail/RedirectScholar?flag=TitleLink&tablename=GARJ2017&filename=SJES1FE3D008D08A03BAE10AD66EDECA67D6) and *M.officinalis.*

**Supplementary Fig 12**. GO enrichment and classification of PD(a) and TD(b) genes in [*M.hypoleuca*](https://kns.cnki.net/KNS8/Detail/RedirectScholar?flag=TitleLink&tablename=GARJ2017&filename=SJES1FE3D008D08A03BAE10AD66EDECA67D6).

# Supplementary Fig s and Tables

For more information on Supplementary Material and for details on the different file types accepted, please see [here](https://www.frontiersin.org/guidelines/author-guidelines#supplementary-material).

## Supplementary Fig s


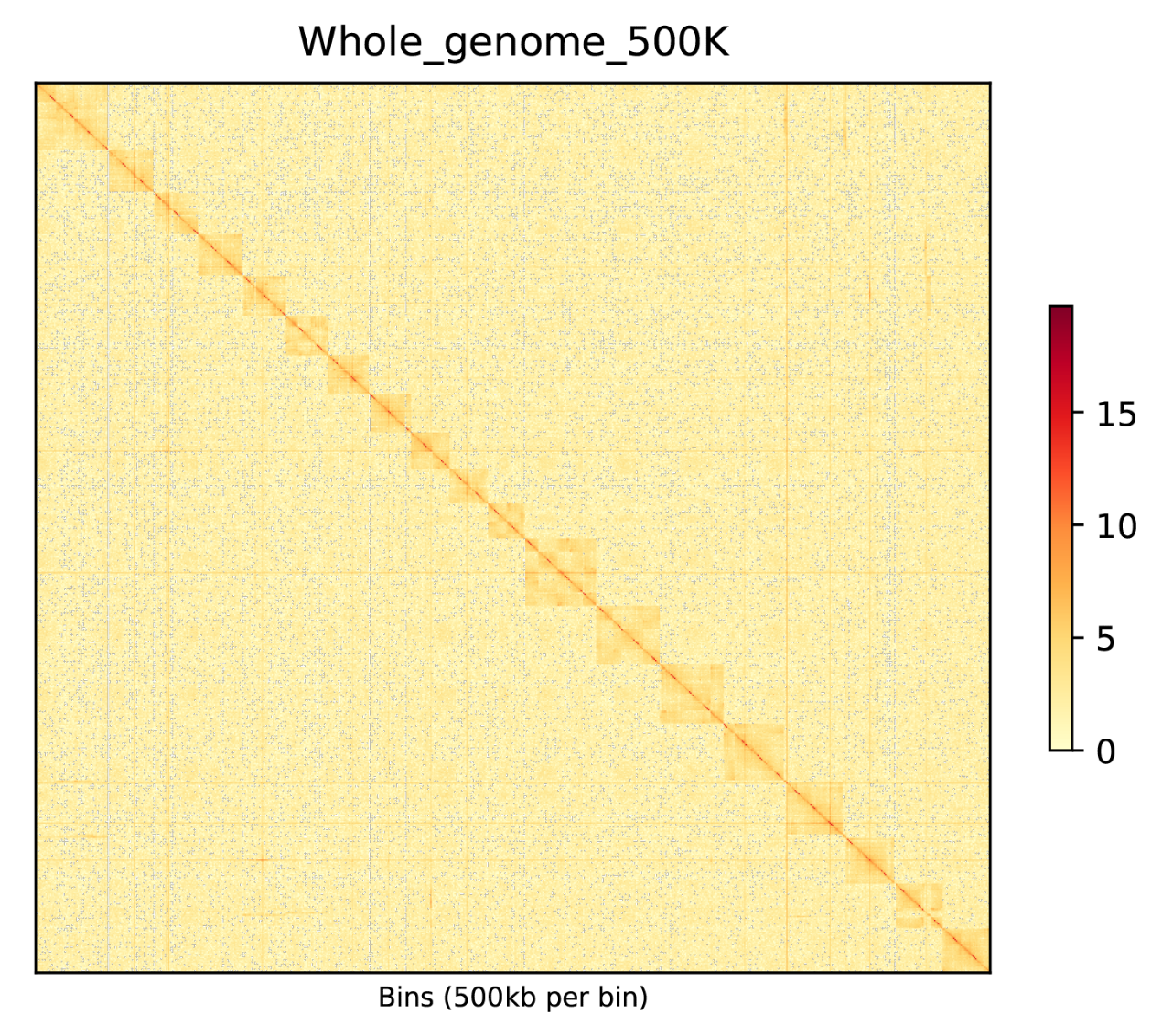


**Supplementary Fig 1**. Automatic Hi-C map of the *M.hypoleuca* genome showing genome-wide all-by-all interactions. The map shows a high resolution of individual chromosomes that are scaffolded and assembled independently. The heat map colors ranging from light yellow to dark red indicate the frequency of Hi-C interaction links from low to high (0–10).


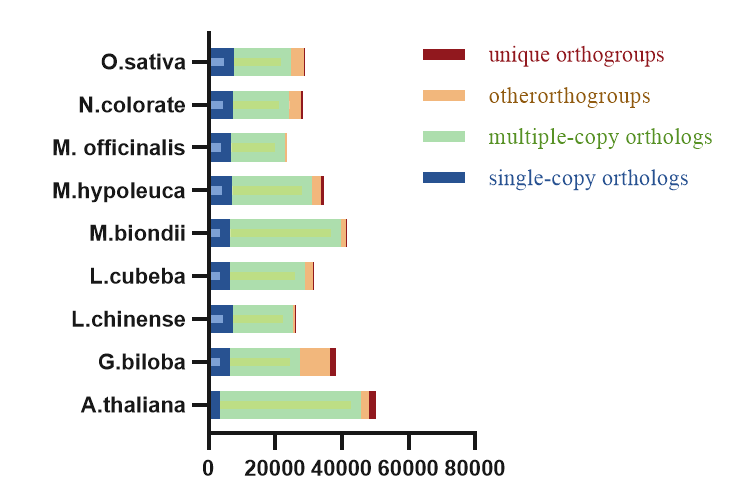


**Supplementary Fig 2**. The distribution of single-copy, multiple-copy, unique, and other orthologs in the 10 plant species.


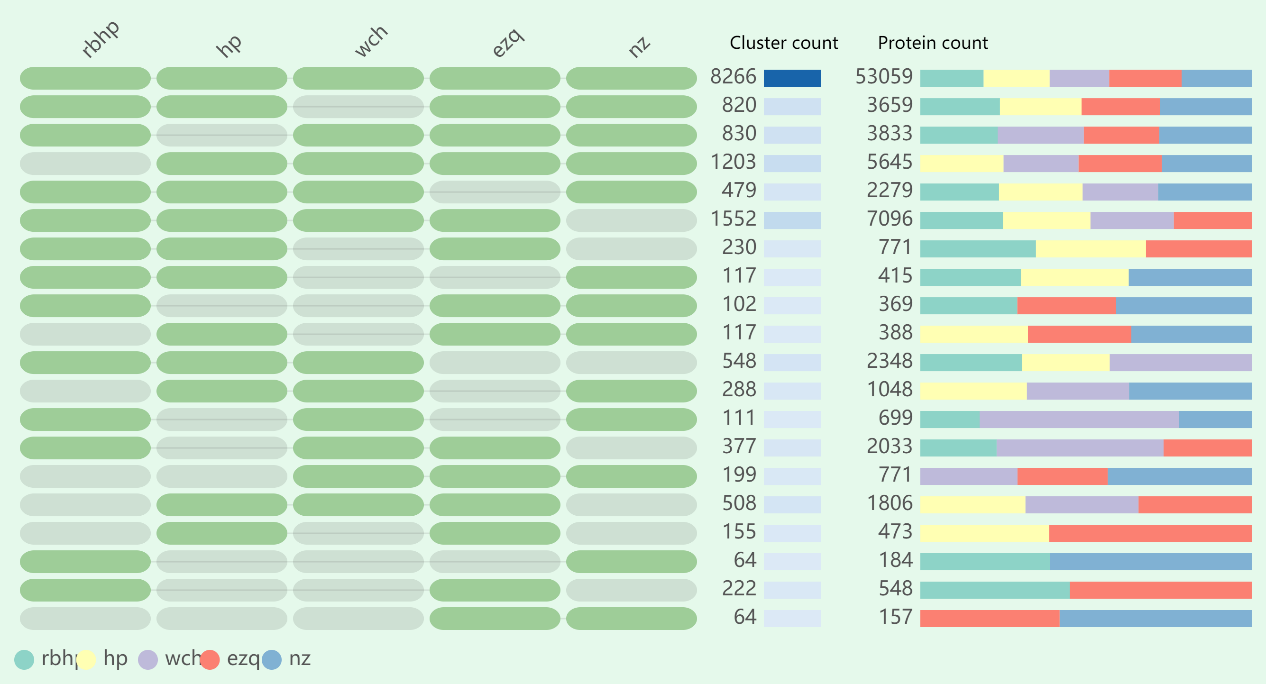


**Supplementary Fig 3**. Sharing of gene families by *M.hypoleuca* and four other species. Cluster count is the number of clusters shared between species. (rbhp: *M.hypoleuca,* hp: *M.officinalis,* wch: *M.biondii,* ezq: *L.chinense,* nz: *C.camphora* )


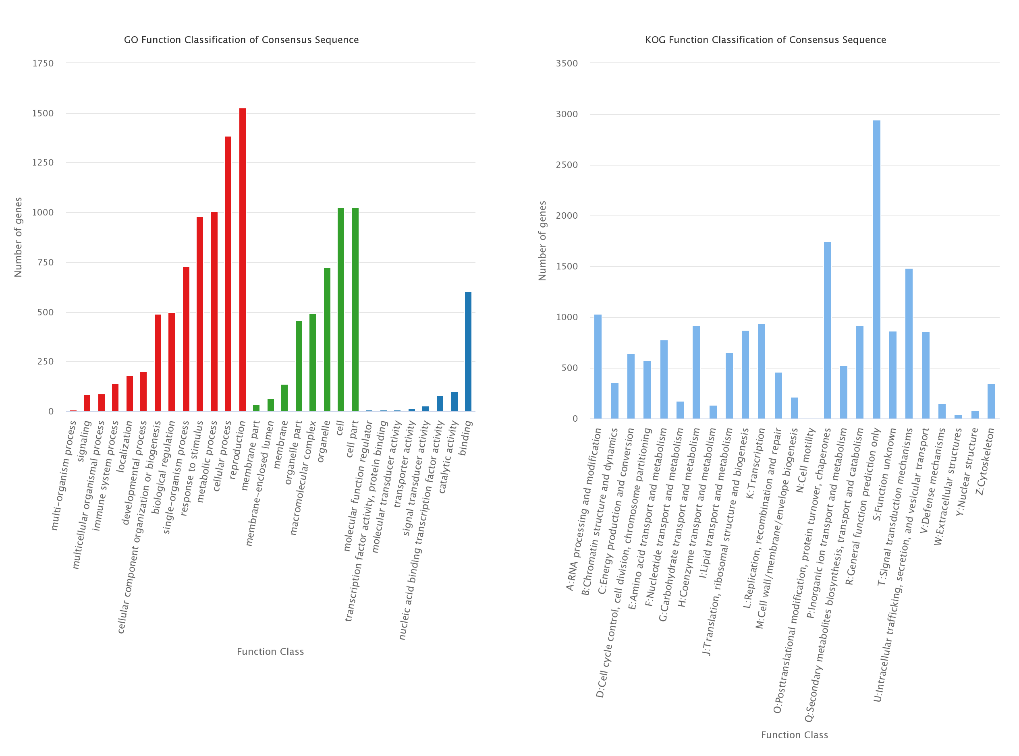


**Supplementary Fig 4**. GO and KOG [function annotation](javascript:;) of protein sequence in *M.hypoleuca.*

**
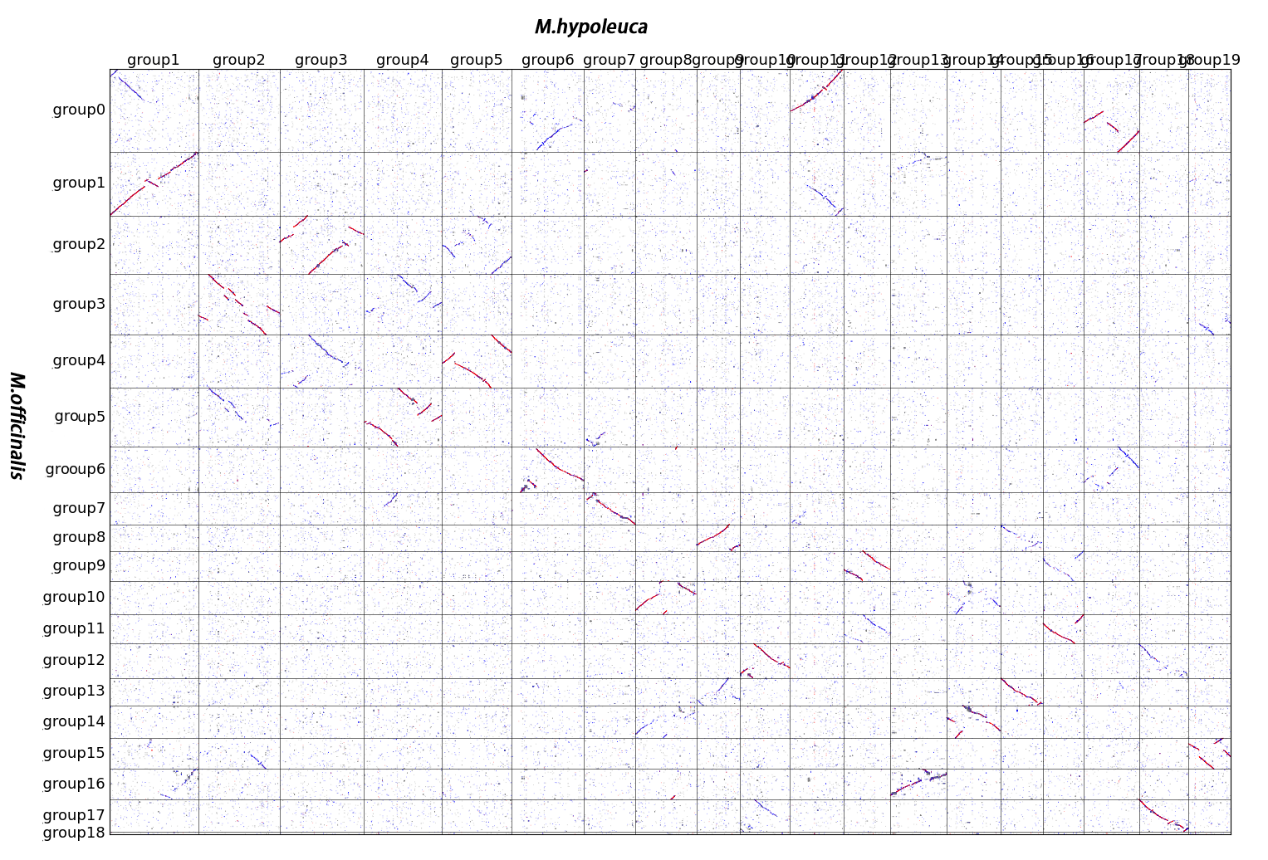
**

**Supplementary Fig 5**. Comparison of [*M.hypoleuca*](https://kns.cnki.net/KNS8/Detail/RedirectScholar?flag=TitleLink&tablename=GARJ2017&filename=SJES1FE3D008D08A03BAE10AD66EDECA67D6) *and M.officinalis* genomes. Dot plots of orthologs showing a 1–1 chromosomal relationship between the [*M.hypoleuca*](https://kns.cnki.net/KNS8/Detail/RedirectScholar?flag=TitleLink&tablename=GARJ2017&filename=SJES1FE3D008D08A03BAE10AD66EDECA67D6) *and M.officinalis* genome.

**
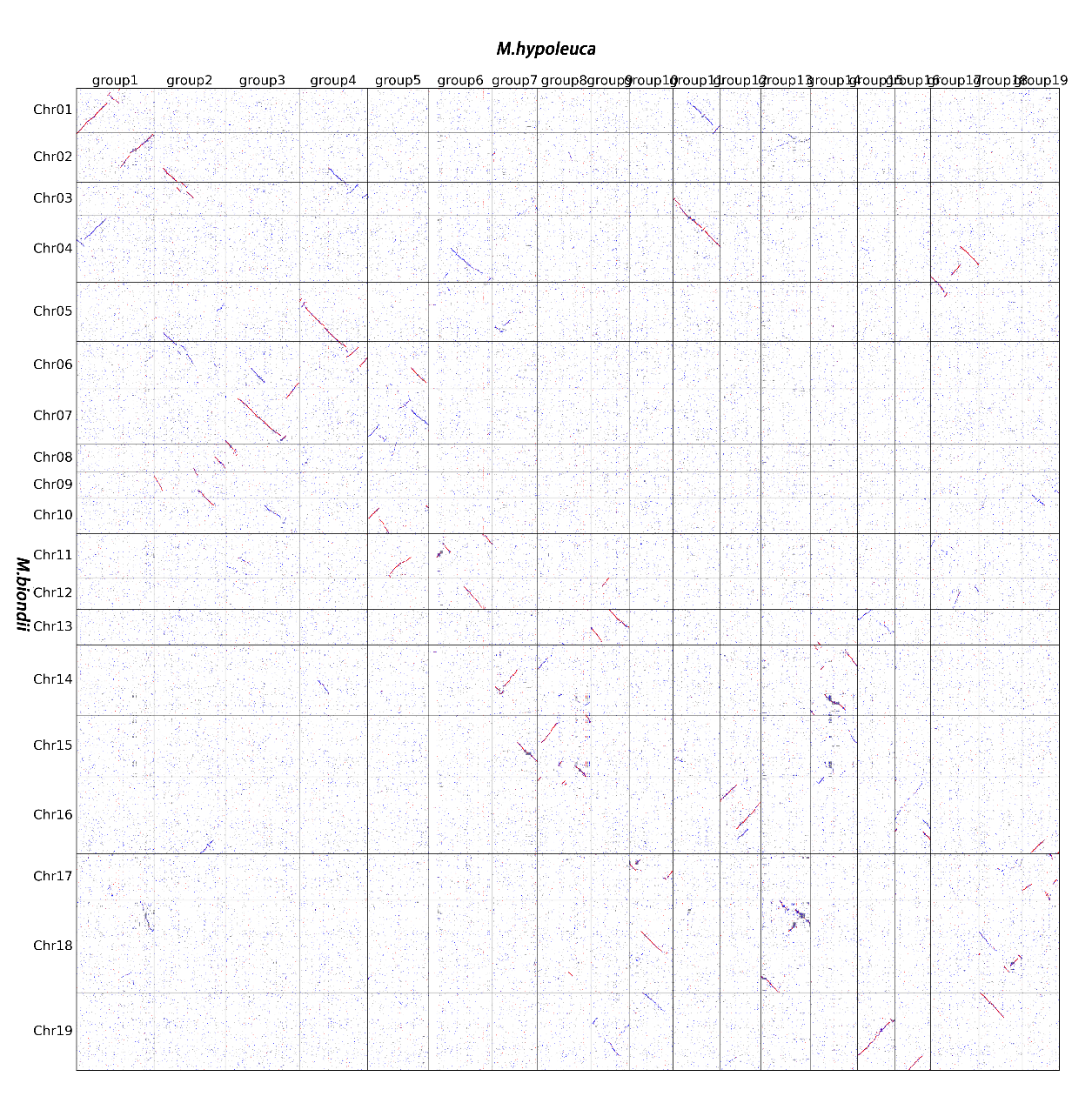
**

**Supplementary Fig 6**. Comparison of [*M.hypoleuca*](https://kns.cnki.net/KNS8/Detail/RedirectScholar?flag=TitleLink&tablename=GARJ2017&filename=SJES1FE3D008D08A03BAE10AD66EDECA67D6) *and M.biondii* genomes. Dot plots of orthologs showing a 1–1 chromosomal relationship between the [*M.hypoleuca*](https://kns.cnki.net/KNS8/Detail/RedirectScholar?flag=TitleLink&tablename=GARJ2017&filename=SJES1FE3D008D08A03BAE10AD66EDECA67D6) *and M.biondii* genome.

**
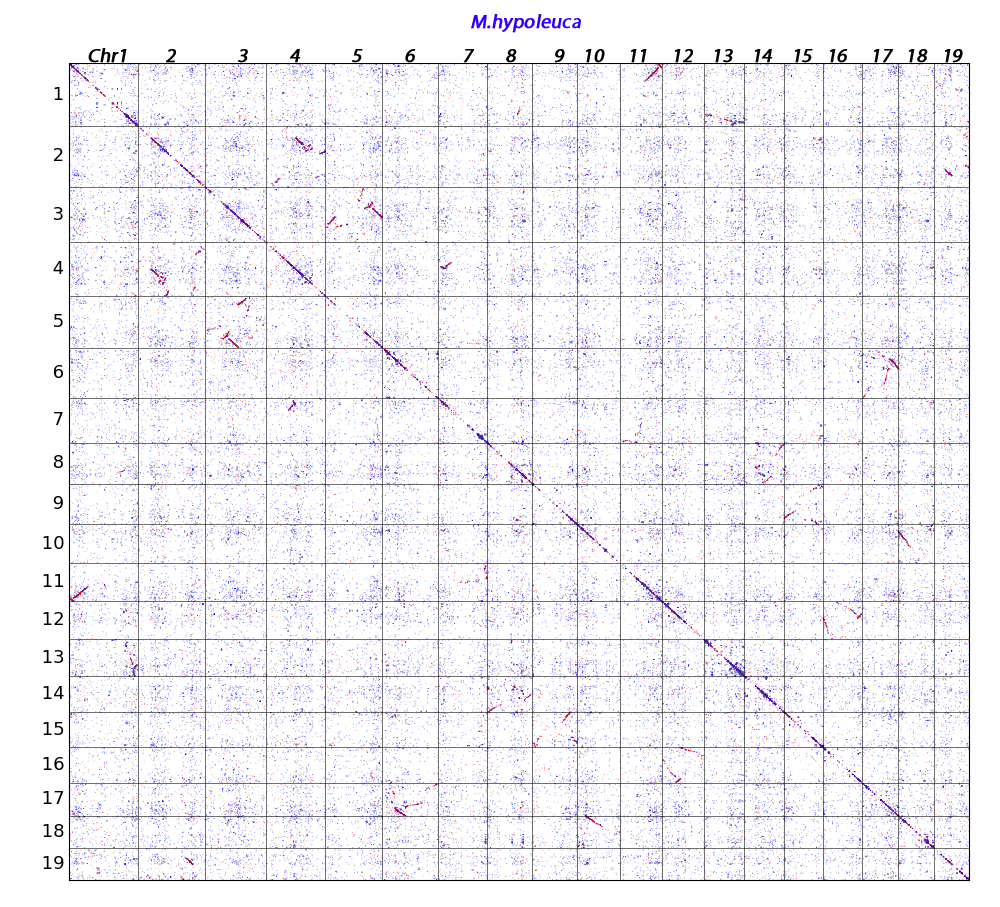
**

**Supplementary Fig 7**. Self-collinearity map of [*M.hypoleuca*](https://kns.cnki.net/KNS8/Detail/RedirectScholar?flag=TitleLink&tablename=GARJ2017&filename=SJES1FE3D008D08A03BAE10AD66EDECA67D6). The values on the X- and Y-axes are the numbers of cumulative genes on the 19 chromosomes.

*
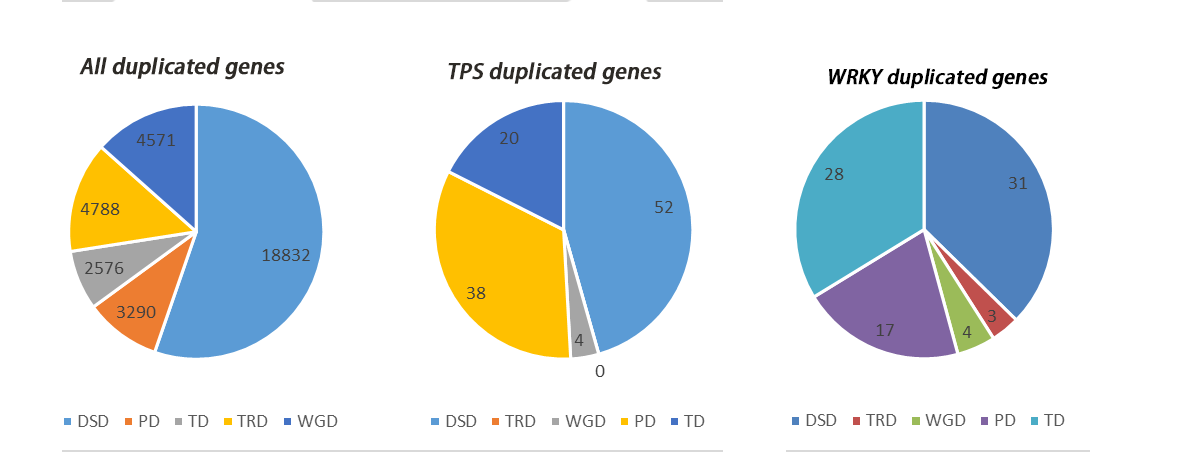
*

**Supplementary Fig 8**. Categories and proportions of different types of duplicated genes in *M. hypoleuca* and *MhTPS, MhWRKY*. TD, tandem duplication; PD, proximal duplication; TRD, transposed duplication; DSD,dispersed duplication.

*
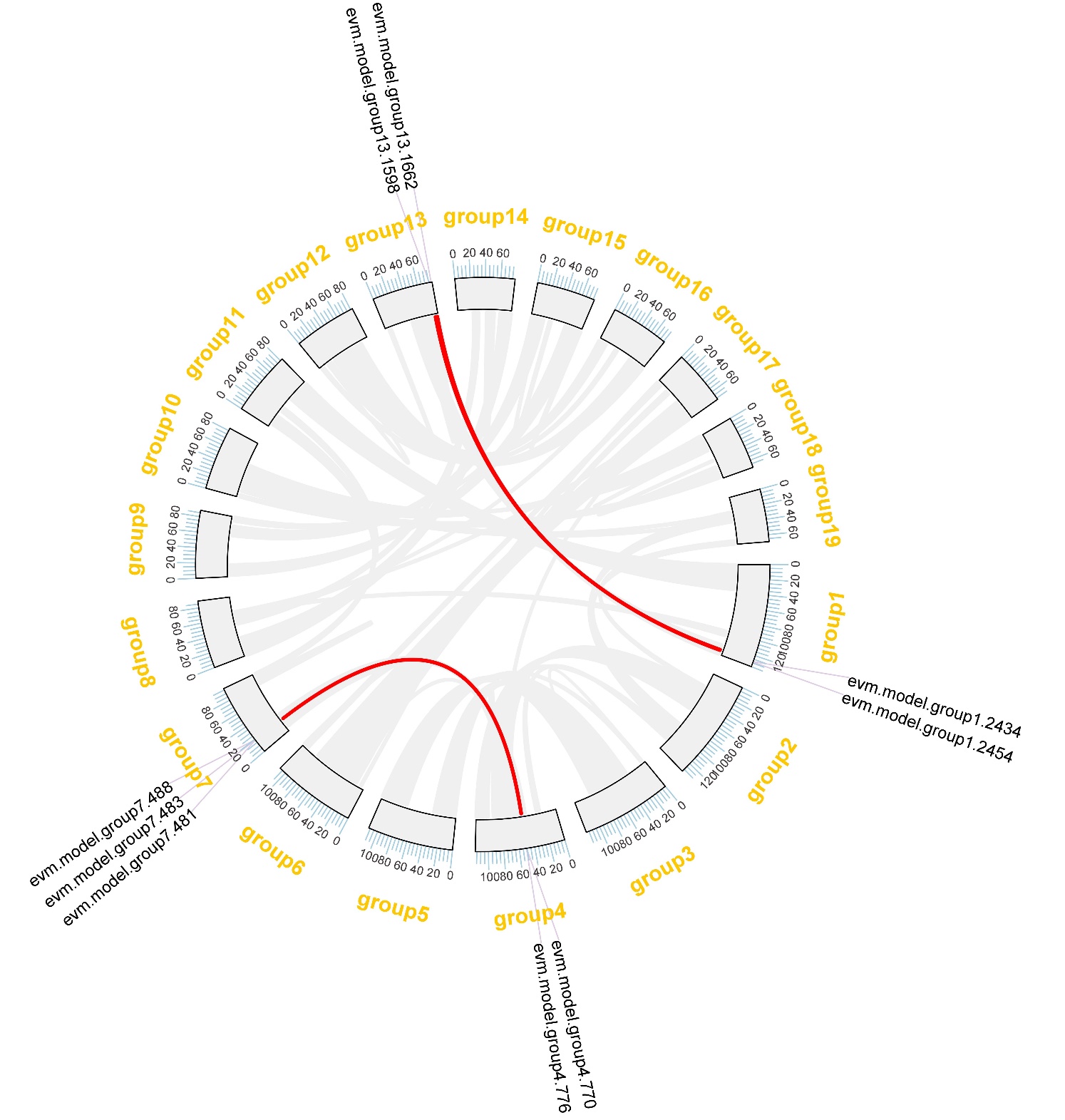
*

**Supplementary Fig 9.** Intergenomic synteny blocks among 9 *TPS* genes of [*M.hypoleuca*](https://kns.cnki.net/KNS8/Detail/RedirectScholar?flag=TitleLink&tablename=GARJ2017&filename=SJES1FE3D008D08A03BAE10AD66EDECA67D6).


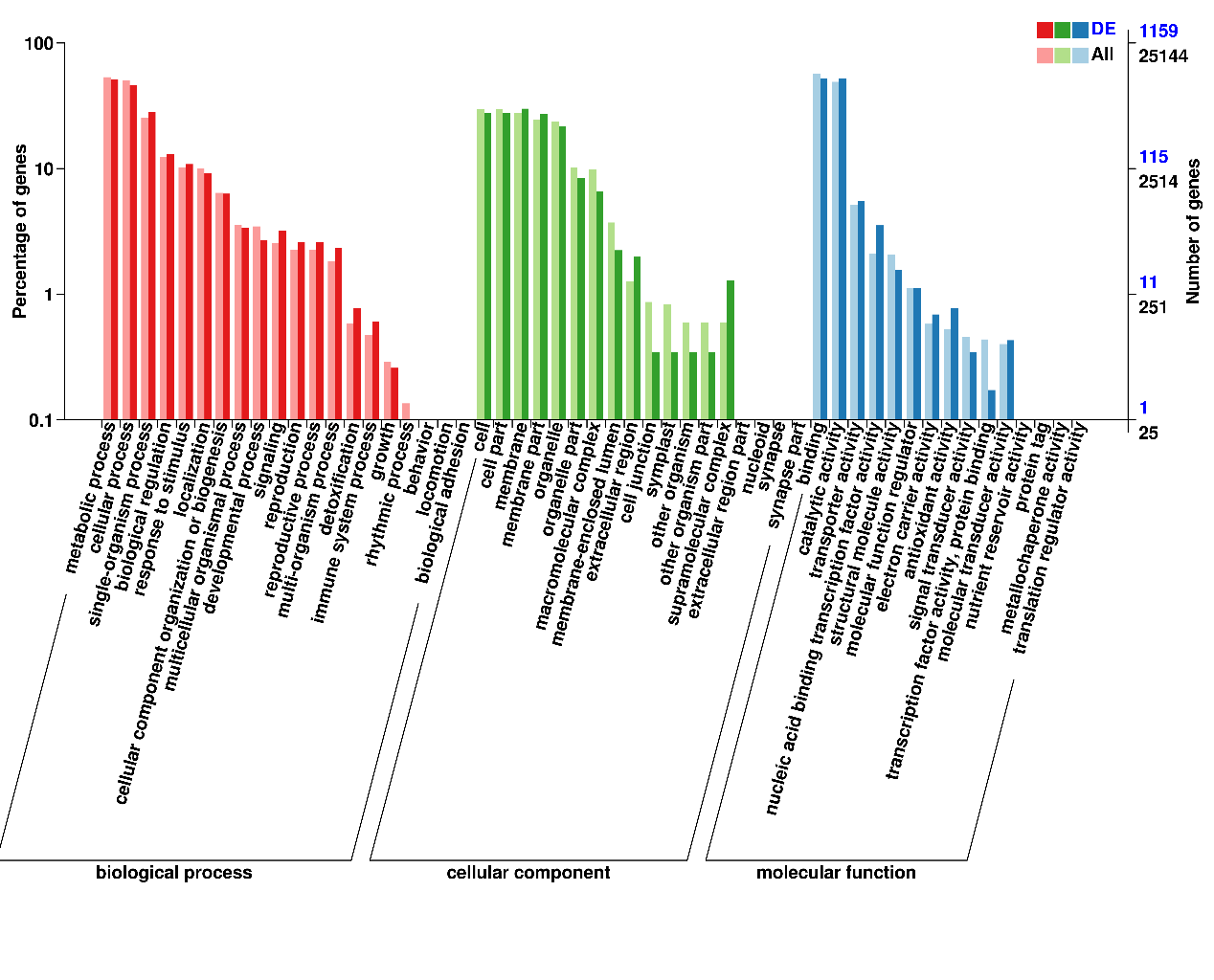


**Supplementary Fig 10**. All Unigene of *[M.hypoleuca](https://kns.cnki.net/KNS8/Detail/RedirectScholar?flag=TitleLink&tablename=GARJ2017&filename=SJES1FE3D008D08A03BAE10AD66EDECA67D6)* and *M.officinalis* and GO classification of DEG


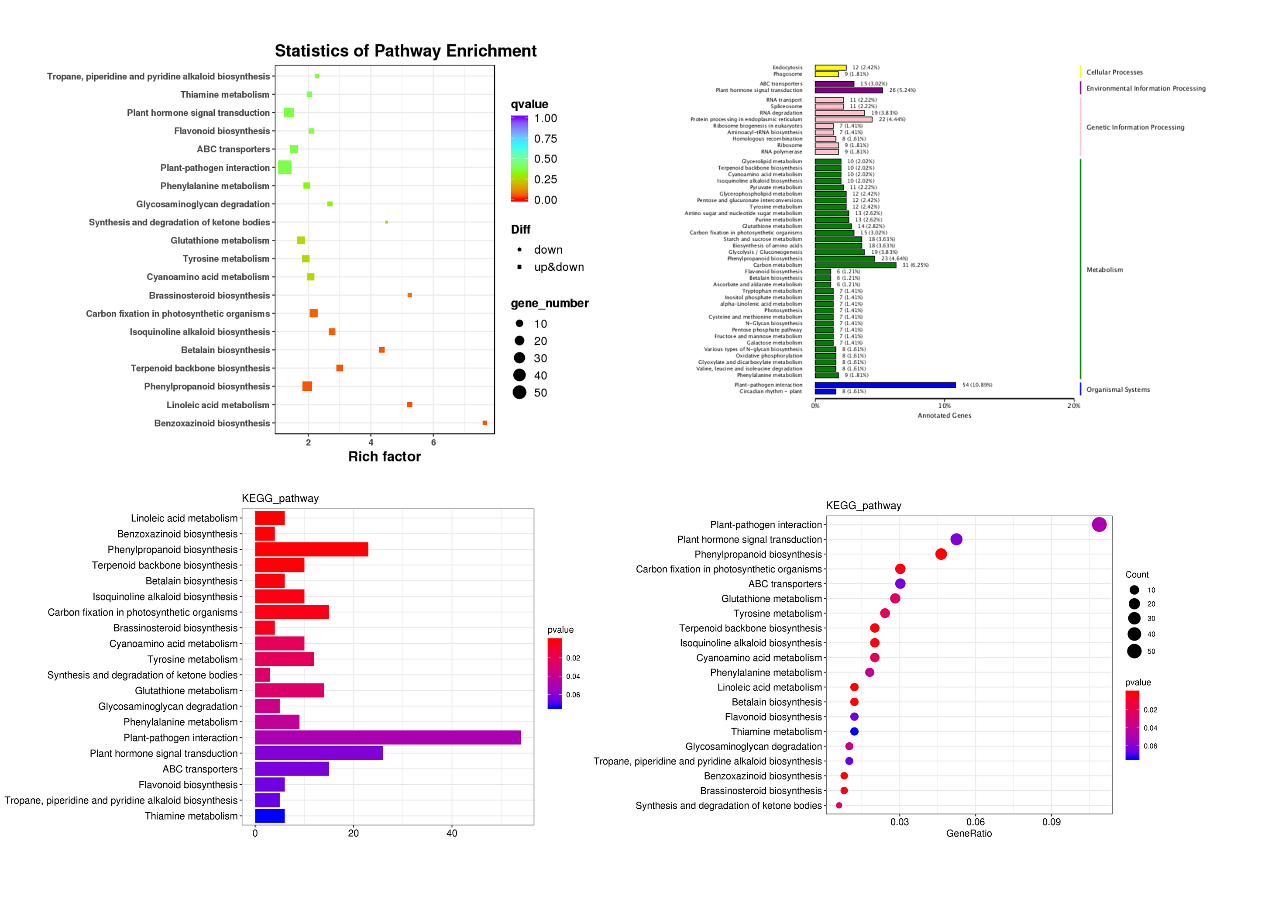


**Supplementary Fig 11**. KEGG enrichment and classification of differentially expressed genes in [*M.hypoleuca*](https://kns.cnki.net/KNS8/Detail/RedirectScholar?flag=TitleLink&tablename=GARJ2017&filename=SJES1FE3D008D08A03BAE10AD66EDECA67D6) and *M.officinalis.*


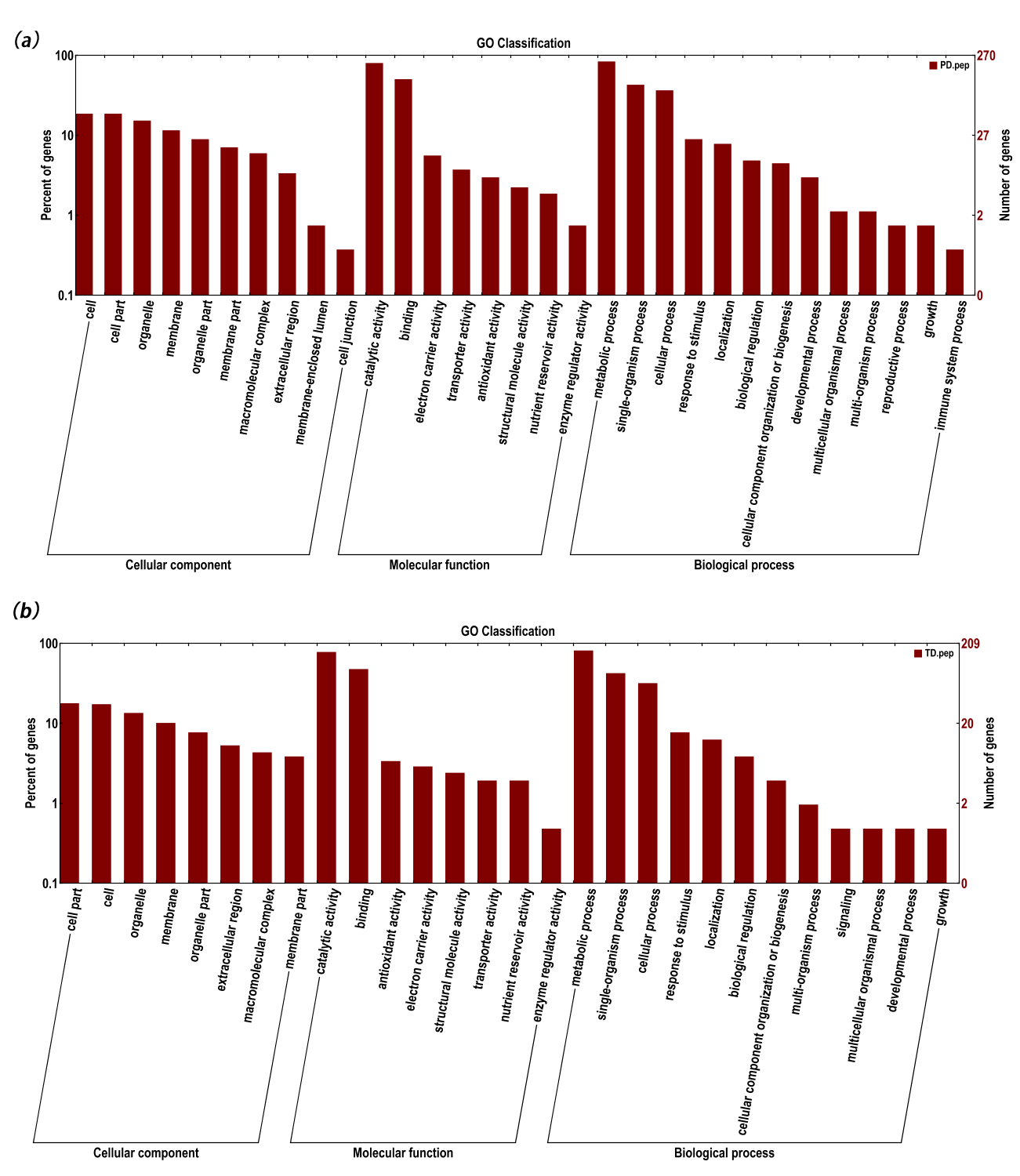


**Supplementary Fig 12**. GO enrichment and classification of PD(a) and TD(b) genes in [*M.hypoleuca*](https://kns.cnki.net/KNS8/Detail/RedirectScholar?flag=TitleLink&tablename=GARJ2017&filename=SJES1FE3D008D08A03BAE10AD66EDECA67D6).
